# Nanomaterials Based on 2,7,12,17-Tetra-tert-butyl-5,10,15,20-tetraaza-21H,23H-porphine Exhibiting Bifunctional Sensitivity for Monitoring Chloramphenicol and Co^2+^

**DOI:** 10.3390/biomedicines12040770

**Published:** 2024-03-30

**Authors:** Ionela Fringu, Diana Anghel, Ion Fratilescu, Camelia Epuran, Mihaela Birdeanu, Eugenia Fagadar-Cosma

**Affiliations:** 1 Institute of Chemistry “Coriolan Dragulescu”, Mihai Viteazu Avenue 24, 300223 Timisoara, Romania; mcreanga@acad-icht.tm.edu.ro (I.F.); danghel@acad-icht.tm.edu.ro (D.A.); ionfratilescu@acad-icht.tm.edu.ro (I.F.); ecamelia@acad-icht.tm.edu.ro (C.E.); 2 National Institute for Research and Development in Electrochemistry and Condensed Matter, P. Andronescu Street, No. 1, 300224 Timisoara, Romania; mihaelabirdeanu@gmail.com

**Keywords:** azaporphyrin, chloramphenicol detection, Co^2+^ detection, UV–Vis, fluorescence, IR

## Abstract

Monitoring antibiotic retention in human body fluids after treatment and controlling heavy metal content in water are important requirements for a healthy society. Therefore, the approach proposed in this study is based on developing new optical sensors using porphyrin or its bifunctional hybrid materials made with AuNPs to accomplish the accurate detection of chloramphenicol and cobalt. To produce the new optical chloramphenicol sensors, 2,7,12,17-tetra-tert-butyl-5,10,15,20-tetraaza-21H,23H-porphine (TBAP) was used, both alone in an acid medium and as a hybrid material with AuNPs in a water–DMSO acidified environment. The same hybrid material in the unchanged water–DMSO medium was the sensing material used for Co^2+^ monitoring. The best results of the hybrid materials were explained by the synergistic effects between the TBAP azaporphyrin and AuNPs. Chloramphenicol was accurately detected in the range of concentrations between 3.58 × 10^−6^ M and 3.37 × 10^−5^ M, and the same hybrid material quantified Co^2+^ in the concentration range of 8.92 × 10^−5^ M–1.77 × 10^−4^ M. In addition, we proved that AuNPs can be used for the detection of azaporphyrin (from 2.66 × 10^−5^ M to 3.29 × 10^−4^ M), making them a useful tool to monitor porphyrin retention after cancer imaging procedures or in porphyria disease. In conclusion, we harnessed the multifunctionality of this azaporphyrin and of its newly obtained AuNP plasmonic hybrids to detect chloramphenicol and Co^2+^ quickly, simply, and with high precision.

## 1. Introduction

Azaporphyrins are used in many modern applications, such as photodynamic therapy for cancer [1,2], light harvesting [3], and sensors [4]. The azaporphyrin 2,7,12,17-tetra-tertbutyl-5,10,15,20-tetraaza-21H,23H-porphine (TBAP), known as a molecular organic semiconductor, has been intensively studied [5,6] because its thin films were reported to have a band gap of 1.75 eV, thus making them suitable for optoelectronic applications [7]. In this work, the TBAP azaporphyrin will be used to develop new UV–vis sensors for chloramphenicol and Co^2+^ monitoring.

The resistance of bacterial strains to extensively used antibiotics has generated a justified concern regarding a return to old treatment options based on chloramphenicol [8]. Chloramphenicol is well known as a very efficient antibiotic inhibiting both Gram-positive and Gram-negative bacteria [9].

Nevertheless, the harmful side effects of chloramphenicol (CHL), such as neurotoxicity and hematologic disorders [10], irreversible aplastic anemia [11], reversible bone marrow suppression [12], and acute myeloid leukemia [13], warrant the careful monitoring of chloramphenicol levels in human biological fluids. Fortunately, bone marrow suppression is a reversible side effect after treatment with chloramphenicol. Chloramphenicol only produces a fall in hemoglobin levels if it reaches a concentration higher than 25 mg/mL in plasma [14].

When large doses of chloramphenicol are given to premature neonates, it causes a severe illness named “gray baby syndrome” that begins 2–9 days after treatment is started. A similar “gray syndrome” has been encountered in adults who have overdosed on this antibiotic [15,16].

Table S1 in the Supplementary Materials presents the most efficient and recently used methods for chloramphenicol detection based on porphyrins or other materials. Despite the fact that Table S1 reports few trace detection domains [17,18], the real purpose of CHL detection is to discover medically relevant concentrations of CHL remaining in the human body after treatment with this medicine. Thus, the real need is to identify toxic concentrations that potentially endanger the patients, that is, a concentration in the range of 1 × 10^−6^ M–5 × 10^−5^ M.

On the other hand, monitoring water and foods regarding their content of heavy metals is a necessity in our society [19,20,21]. Among these metals, cobalt, especially the frequently used cobaltous (Co^2+^) type, although needed as vitamin B12, can be considered to be toxic to humans and the environment at increased levels of exposure [22]. Table S2 from the Supplementary Materials contains data concerning recent performances in Co^2+^ detection. Several methods for the determination of cobalt have been reported, which mainly focused on spectrophotometry [23], flow injection [24], liquid chromatography [25], and capillary electrophoresis [26]. Co^2+^ detection in different sources, such as water, milk, spinach leaves, cabbage leaves, lettuce leaves, parsnip root, celery root, garlic root, white onion root, red onion root, orange, tangerine, red grapefruit, apple, pear, milk, powder milk, chicken liver, flour, cinnamon, coffee, and beer [27], is needed to control the content of this metal in different foods [28]. The highest relevant Co^2+^ concentration is 5.92 × 10^−3^ M [29] in baby milk; it is around 50 µM in water samples of different origins, such as drinking water, lake water, seawater, and river water [30,31], and in the range from 5 to 300 µM in different foods like instant coffee, lamb kidneys, Brazil nuts, chocolate milk, linseeds, brewers’ yeast, millet seeds, buckwheat, kidney beans, dark chocolate, rice, chili powder, sunflower kernels, bovine liver, curry powder, cashew nuts, peanut butter, potatoes, fresh broccoli, and brown lentils. 

By harnessing the multifunctionality and optical capabilities of 2,7,12,17-tetra-tertbutyl-5,10,15,20-tetraaza-21H,23H-porphine (TBAP), both alone and of its hybrid materials obtained with AuNPs in acidic and organic media, this study focuses on developing two new simple methods for the detection of an antibiotic, chloramphenicol, and of cobalt, the latter being appropriate for monitoring the water environment. Our approach is original and based on new plasmonic materials; it is also fast and simply synthesized by complexing the TBAP azaporphyrin with AuNPs.

## 2. Materials and Methods

### 2.1. Materials

The 2,7,12,17-Tetra-tert-butyl-5,10,15,20-tetraaza-21H,23H-porphine (TBAP) was purchased from Sigma-Aldrich^®^ (Darmstadt, Germany); chloramphenicol (CHL) was acquired from Northeast Pharmaceutical, Group Co. (Shenyang, China); HAuCl_4_ × 3H_2_O was supplied by Roth (Karlsruhe, Germany); cobalt(II)acetate, HCl, NaOH, and dimethyl sulfoxide (DMSO) were bought from Merck (Darmstadt, Germany); niacin, lactic acid (LA), BaCl_2_, MnCl_2_, NaCl, calcium gluconate (CaGlu), SnCl_2_, β-Carotene, and KI were obtained from Merck (Darmstadt, Germany); FeCl_3_ was provided by Fluka Chemie (Buchs, Switzerland); glucose (Glu) was provided by Chimreactiv/Reactivul (Bucuresti, Romania), calcium lactate (CaL) was purchased from DH Laboratory Chemicals (Poole, UK). All the reagents of *purrum analyticum grade* were used as received, without further purification.

### 2.2. Apparatus

In order to record the UV–Vis spectra (1 cm wide quartz cuvettes), a V-650 JASCO spectrometer (Pfungstadt, Germany) was used. For atomic force microscopy (AFM) images, a Nanosurf^®^ EasyScan 2 Advanced Research AFM microscope (Liestal, Switzerland) was used, equipped with a piezoelectric ceramic cantilever. The AFM measurements were performed in ambient conditions at a temperature of 21 ± 2 °C (relative humidity: 50–70%) in contact mode. The samples were prepared by drop-casting from the DMSO or DMSO/water solutions. The emission spectra were recorded on a Perkin–Elmer Model LS 55 apparatus (Waltham, MA, USA), using 1 cm path length cells, a scan speed of 100 nm/min, excitation of λ = 364 nm, excitation slits of 10 nm, emission slits of 5 nm, at ambient temperature (22–24 °C), without cut-off filters. To register FT-IR spectra, KBr pellets or the ATR mode were used on a JASCO 430 FT-IR (Hachioji, Tokyo, Japan) spectrometer, working in the range 4000–400 cm^−^^1^. A 400 MHz Bruker Avance NEO Spectrometer (Rheinsteitten, Germany) equipped with 5 mm four nuclei (^1^H/^13^C/^19^F/^29^Si) provided the ^1^H-NMR spectra, registered in CDCl_3_. The chemical shifts are expressed in ppm, using tetramethylsilane (TMS) as a reference. The interaction mechanisms between TBAP, CHL, and Co^2+^ were plotted by UCSF Chimera (software version 1.16) using a Structure Analysis tool, FindHBond, and electrostatic surface analysis, respectively. Error bars were calculated using the standard deviation of three individual experiments in an Excel worksheet [32,33]. A Titan G2 80–200 TEM/STEM microscope (FEI Company, Eindhoven, The Netherlands) was used in TEM and STEM modes at 80 and 200 kV acceleration voltages, using Digital Micrograph v. 2.12.1579.0 and TEM Imaging and Analysis v.4.7 software. TEM/STEM samples preparations were performed as follows: gold colloid solutions were deposited by drop casting on TEM copper grids covered with carbon film.

## 3. Results and Discussion

### 3.1. UV–Vis Study Regarding 2,7,12,17-Tetra-tert-butyl-5,10,15,20-tetraaza-21H,23H-porphine (TBAP)

**Figure 1:**
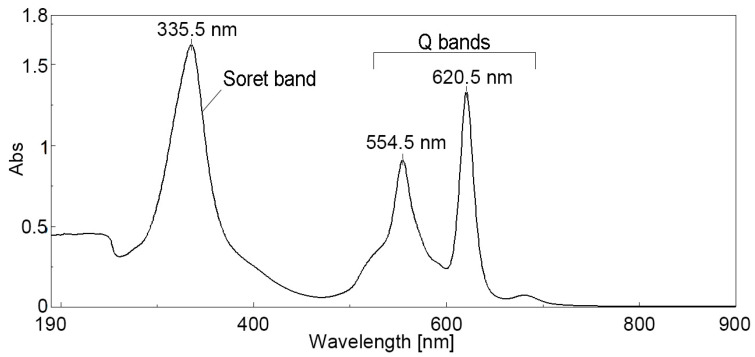
UV–Vis spectra of 2,7,12,17-Tetra-tert-butyl-5,1-,15,20-tetraaza-21H,23H-porphine (TBAP) in DMSO (c = 6.691 × 10^−5^ M); λ_max_(logε): 335.5 (4.383); 355.5 (4.132); 620.5 (4.297).

The UV–Vis spectra of the TBAP in DMSO solution, presented in Figure 1, contain the typical Soret around 330 nm and two Q-bands in the ranges of 550−630 nm, respectively. The Soret band, displayed at 335.5 nm, exhibits higher molar coefficients due to π-conjugation. This azaporphyrin has a D_2h_ symmetry, and as expected, shows the splitting of the Q-band, due to its lower symmetry.

The Q band is associated with π−π* HOMO-LUMO doubly degenerated transition from the ground state of a_1u_ symmetry to the first excited state, which is of e_g_ symmetry. A Q_x_ band that is read at 620.5 nm is attributed to a single electronic transition and has higher intensity than Q_y_ band, read at 554.5 nm, that might be attributed to vibronic transitions [7,34,35,36]. The last Q band located around 680 nm can be attributed to potential J-type aggregation [37].

In comparison with usual porphyrins, in TBAP azaporphyrin, the highest occupied molecular orbital (HOMO) and lowest unoccupied molecular orbital (LUMO) energy levels are significantly stabilized by introducing nitrogen atoms instead of carbon atoms at the meso-positions [38,39]. The stabilization degree is higher in the LUMO than in the HOMO, which decreases the HOMO–LUMO gap [40].

Extended UV–Vis studies regarding aggregation for TBAP in DMSO solution at different concentrations (Figure S1) and its optical behavior in acid-based media (Figure S2) and in base medium (Figure S3) are presented in the Supplementary Materials.

### 3.2. Fluorescence Study of 2,7,12,17-Tetra-tert-butyl-5,10,15,20-tetraaza-21H,23H-porphine (TBAP)

The capacity of porphyrins to release fluorescence makes them desired compounds for imaging diagnostics, especially for tumors [41]. As can be seen from Figure S4, the TBAP azaporphyrin demonstrates intense fluorescence, looking like a light-red glowing material during exposure to λ = 366 nm [42].

**Figure 2:**
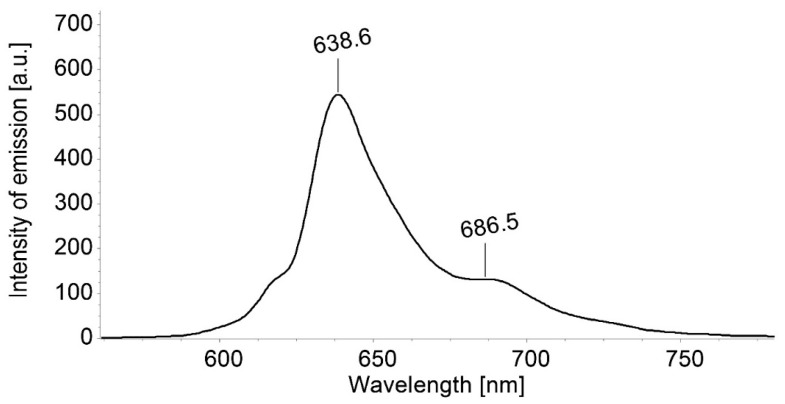
Emission spectra for a solution of TBAP in DMSO (c = 3.941 × 10^−5^ M); excitation wavelength: 364 nm, excitation slit of 10 nm, emission slit of 5 nm, and no filter. The scanning rate was 100 nm/min.

**Figure 3:**
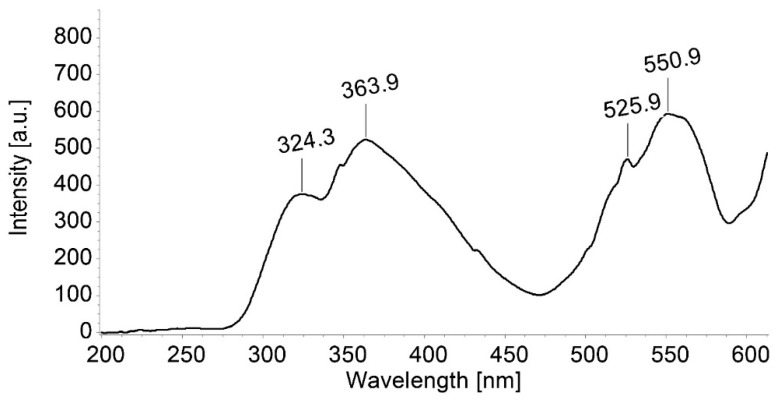
Excitation spectra for a solution of TBAP in DMSO (c = 3.941 × 10^−5^ M); emission wavelength: 639 nm, excitation slit of 10 nm, emission slit of 5 nm, and no filter. The scanning rate was 100 nm/min.

The emission and excitation spectra of TBAP registered in DMSO solutions are given in Figure 2 and Figure 3.

The emission spectrum of TBAP azaporphyrin in DMSO (Figure 2) is characterized by a strong band with the highest maximum around 640 nm. This intense Q(0,0) band is accompanied by a less intense Q(1,0) band at 686 nm, thus mirroring the shape of Q-bands from the absorption spectrum.

**Figure 4:**
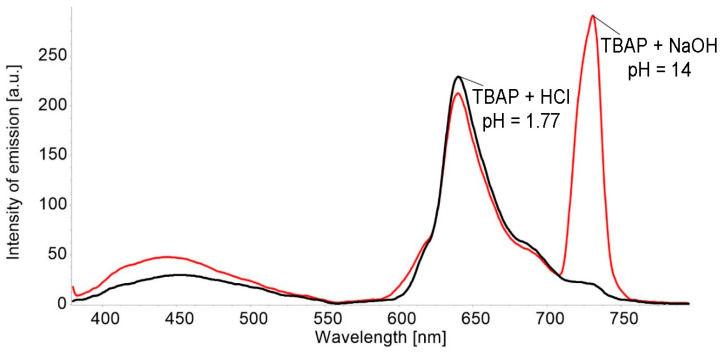
Compared emission spectra for a solution of acidulated TBAP to pH = 1.77 and a basic solution of TBAP (pH = 14), in both DMSO (c = 3.941 × 10^−5^ M), λ_ex_ = 364 nm; excitation slit of 10 nm, emission slit of 5 nm, and no filter. The scanning rate was 100 nm/min.

**Figure 5:**
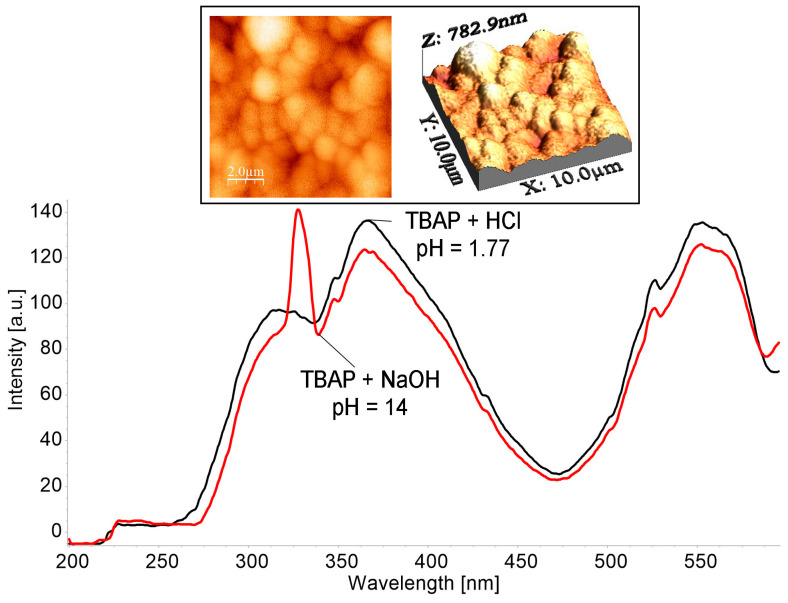
Comparison of excitation spectra between a solution of acidulated TBAP to pH = 1.77 and a basic solution of TBAP (pH = 14), in both DMSO (c = 3.941 × 10^−5^ M), λ_em_ = 639 nm; excitation slit of 10 nm, emission slit of 5 nm, and no filter. The scanning rate was 100 nm/min; 2D and 3D images recorded in AFM (contact mode) of supramolecular architectures created by TBAP in DMSO solution at pH = 14 are presented in detail.

A comparison of the emission and excitation spectra at different pH of DMSO solutions are presented in Figure 4 and Figure 5.

The significant increase in the intensity of the Q band located around 750 nm in the solution environment at pH = 14 can be explained due to symmetry changes in the azaporphyrin core; these are caused by the interactions with the OH- groups from the base [43].

The band around 750 nm, displayed in Figure 4, might also be explained by significant orbital mixing and excited stated properties that are solvent dependent and that also imply torsional motions and oscillator strength of the S_0_→S_1_ transition. In addition, the molecule axes might not coincide with the directions of the polarization of the S_0_→S_1_ transition [44]. All these contributions might produce a redistribution of intensity; thus, the visible bands now have similar or higher intensity compared with the band located around 430 nm [45].

The distinct peak on emission spectrum, shown in Figure 5, located at 320 nm in DMSO solution at pH = 14, is an indication for the well-constructed *H*-type aggregates. These sandwich type aggregates are also confirmed by 2D and 3D AFM microscopy that present large strow-like supramolecular architectures. Helicoidal organization of the corn grains type aggregates is visible in the 2D AFM image and leave room for large voids that are suitable for interactions with different analytes.

The calculated Stokes shift (λ_em_–λ_abs_) [46] for acid solutions is 299 nm and the Stokes shift for basic solution is a little higher at 304 nm. The large Stokes shifts, both in basic and acid solutions, might indicate that the structures of porphyrin are different in the excited state as compared with the ground state. A reorganization of the porphyrin electronic state and its geometry might occur in the excited state, producing the distortion of the planar structure. A large Stokes shift thus suggests better fluorescence properties because the shift allows for the easy separation of the emission from scattered light, indicating a small resonance energy transfer [47,48].

**Figure 6:**
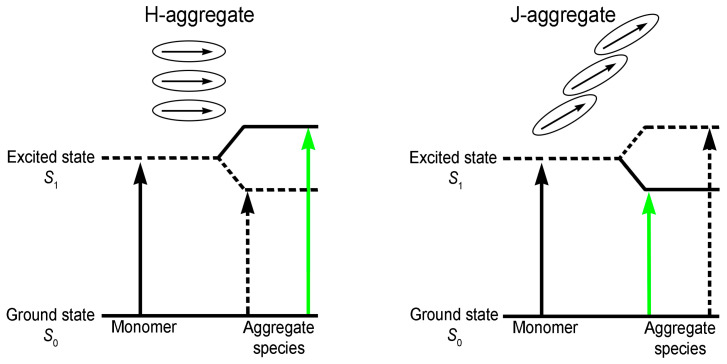
Energy diagram of H and J aggregate transitions.

The energy diagram of H and J aggregates transitions is presented in Figure 6.

In the H-type aggregates, the Soret band is hypsochromically shifted due to a face-to-face arrangement of monomers. Instead, the J-type aggregates show a narrow Soret band red shifted compared with the monomer and the porphyrin monomers are aligned head-to-tail [49].

Thus, only the transition to the higher energetic exciton level is allowed for the H-aggregates. In the case of the J-aggregates [50,51], the only allowed transition is that to the lower energetic state [52,53].

### 3.3. 1H-NMR Study of the TBAP Azaporphyrin

**Figure 7:**
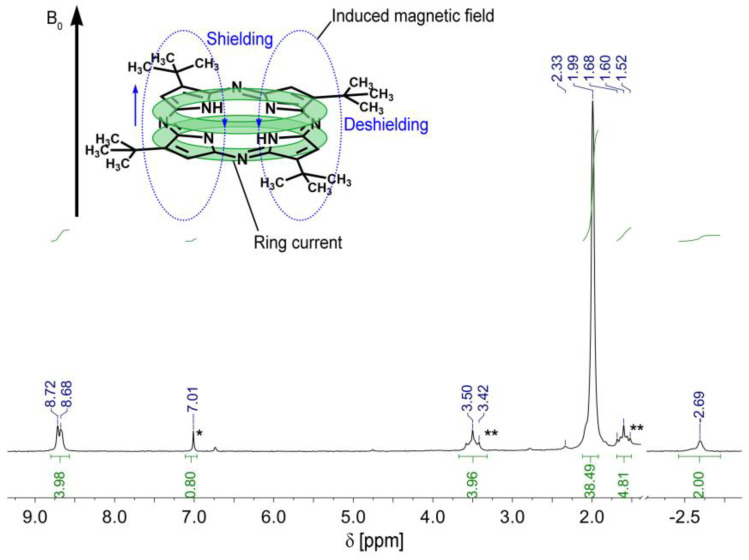
The ^1^H-NMR of TBAP porphyrin in CDCl_3_. One can see the shielding/de-shielding phenomena in detail due to the induced ring current, which is a consequence of the induced magnetic field. The starred marked signals are impurities from the deuterated solvent.

The most important aspects in the ^1^H-NMR spectrum (Figure 7) of the TBAP are the shifting of the signals due to induced magnetic field. The strong shielding effect of internal NH is moving the signal to −2.69 ppm and the strong de-shielding effect of the β-pyrrolic protons, produces a doublet signal between 8.72 and 8.68 ppm, respectively. This shielding/de-shielding phenomenon is caused by the induced ring current as a result of the induced magnetic field [32,54], also known in the literature as an effect of diamagnetic anisotropy [55], manifested when paramagnetic states with nonzero spin multiplicity have magnetic-field-dependent state energies [56].

The strongest signal appearing at 1.99 ppm is attributed to the 36 protons from the tert-butyl peripheral groups (-C***H***_3_).

The singlet resonating at 7.01 ppm* is attributed to non-deuterated chloroform residues from CDCl_3_. Purification of the TBAP sample required extraction with THF; this fact produced the appearance of the two triplet type signals between 3.50 and 3.42 ppm** (4H, -O-C***H***_2_-CH_2_-) and between 1.52 and 1.68 ppm** (4H, O-CH_2_-C***H***_2_-), caused by traces of THF [57].

### 3.4. The Optical Detection of CHL by UV–Vis Spectroscopy Used as a Sensitive Substance, TBAP, in Acid Medium

To the 5 mL 2,7,12,17-tetra-tet-butyl-5,1-,15,20-tetraaza-21H,23H-porphine solution in DMSO (c = 6.691 × 10^−^^5^ M) with 0.1 mL HCl 37% (to obtain a pH = 1.9 from initial pH = 9.5 of porphyrin), 0.1 mL portions of chloramphenicol in H_2_O (c = 5.124 × 10^−^^5^ M) were added. Each mixture was vigorously stirred for 60 s and the UV–Vis spectra were recorded. Chloramphenicol does not absorb in the region of interest for its detection by using an azaporphyrin derivative.

**Figure 8:**
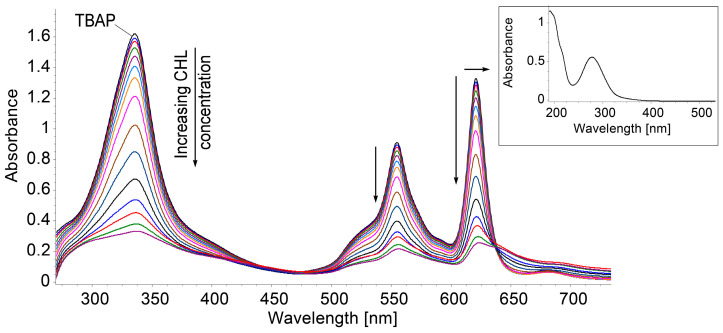
UV–Vis spectra for all samples regarding acidified TBAP in which CHL is added. In detail, UV–Vis spectrum of chloramphenicol (CHL) in water (c = 5.124 × 10^−5^ M).

As displayed in Figure 8 by continuously adding CHL, the intensity of all bands is decreased and an isosbestic point is formed at around 640 nm. There is a good polynomial sigmoidal correlation between the CHL concentration and the absorption intensity of acidulated azaporphyrin, in a narrow range from 3.64 to 10.20 µM; according to [16], this is suitable for the determination of CHL from food products [58].

**Figure 9:**
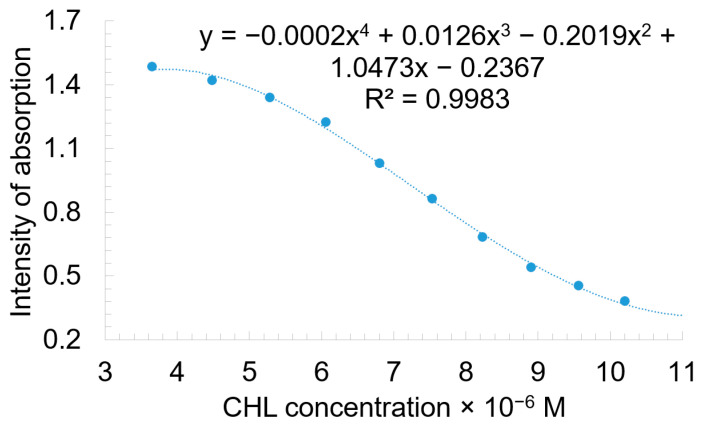
Correlation between CHL concentration and absorption intensity of acidulated TBAP in UV–Vis read at 335.5 nm.

Because the range of detection is so narrow (Figure 9), even if the precision is very high, we developed a plasmonic material based on TBAP to use as a sensitive material for CHL detection in a wide range of concentrations.

### 3.5. The Detection of CHL Using a Plasmonic Complex Formed between Acidified TBAP and AuNPs

#### 3.5.1. Obtaining and Characterization of AuNPs

**Figure 10:**
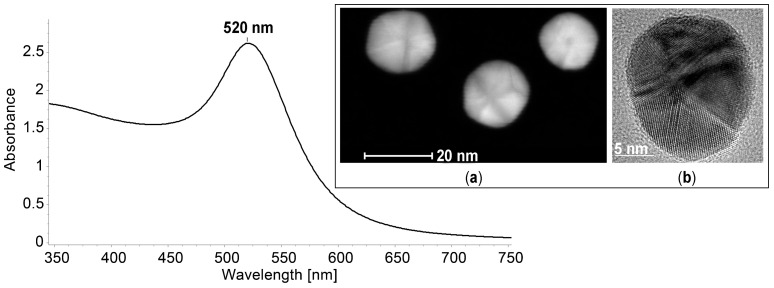
UV–Vis spectra of AuNPs in water (c = 6.85 × 10^−4^ M); (**a**) STEM and (**b**) TEM images of polycrystalline gold nanoparticles.

The synthesis of gold nanoparticles was performed according to the previously reported recipe [59], as follows: 0.035 g HAuCl_4_ × 3H_2_O (0.088 × 10^−3^ mole) dissolved in 116.2 mL doubly distilled water was brought to reflux into a three-necked flask of 150 mL equipped with mechanical stirrer and refrigerator. Then, a 12.25 mL (1 wt %) solution of trisodium citrate (0.122 g, 0.41 × 10^−3^ mole) in doubly distilled water was added at once and the mixture was vigorously stirred and further refluxed for 15 min. The molar ratio of HAuCl_4_/citrate salt was 1:5 [60,61]. Besides acting as reduction agent, the citrate ions also act as stabilizers. The UV–Vis spectrum of AuNPs at a concentration of 6.85 × 10^−4^ M is presented in Figure 10, showing a typical plasmonic alure with a maximum absorption located at 520 nm. The narrowly tailored size of the obtained spherical gold nanoparticles ranges from 15 to 17 nm, as illustrated by the STEM and TEM images in the detail presented in Figure 10.

#### 3.5.2. Method for Obtaining the Complex between TBAP and AuNPs

To 5 mL TBAP in DMSO solution (c = 5.124 × 10^−^^5^ M), 0.1 mL HCl (37%) was added. The resulting pH of the solution was 2. To this obtained solution, different portions of AuNPs (c = 6.85 × 10^−4^ M) were added in the range of 0.05 mL–0.20 mL.

**Figure 11:**
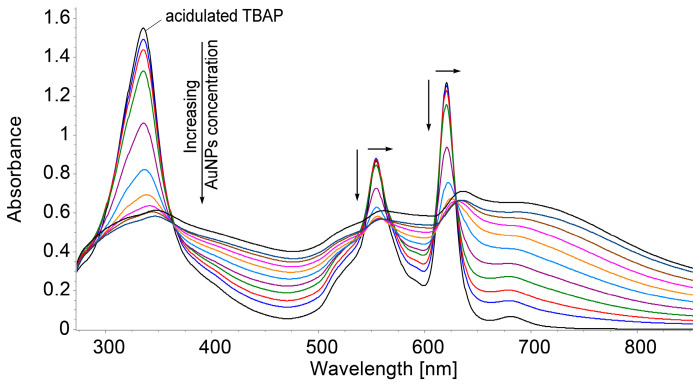
UV–Vis monitoring of AuNPs–TBAP complex formation in DMSO/water system.

As can be seen in Figure 11, the complex between TBAP and AuNPs exhibit an enlarged absorption band from 340 nm to 800 nm; during the complex formation, all the bands of azaporphyrin are slightly red-shifted. The five isosbestic points that are displayed on the UV–Vis spectra (at 375 nm, 535 nm, 566 nm, 612 nm, and 630 nm) demonstrate the existence of some intermediate species between the AuNPs and azaporphyrin that affect the entire macrocycle.

The synergistic effect between AuNPs and porphyrins generally provides an enlarged absorption domain [62,63,64] that is useful for improving optical applications. In the formed complex, the gold nanoparticles that are negatively surface charged easily interact with the partially positively charged nitrogen atoms of the porphyrin core in the porphyrin base, or with the diprotonated porphyrin that forms in the acid medium.

#### 3.5.3. The Optical Detection of CHL Using the Hybrid Material AuNPs–TBAP

To a quantity of 5 mL complex in DMSO/acidified water, portions of 0.05 mL of CHL solution in water (c = 5.12 × 10^−4^ M) were added. After each addition, the mixture was stirred for 90 s and the UV–Vis spectra were recorded.

**Figure 12:**
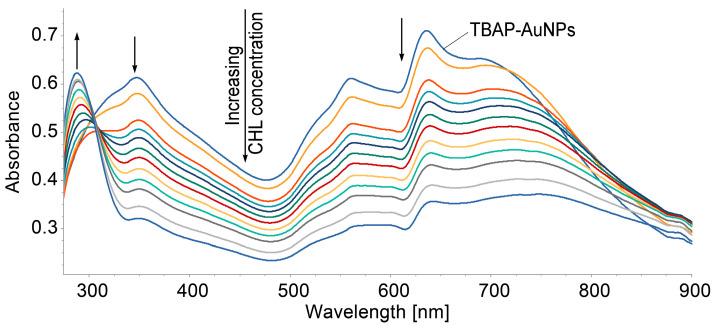
Overlapping UV–Vis spectra of the AuNPs–TBAP complex in DMSO/water after the stepwise addition of CHL.

The addition of CHL to the complex caused a decrease in the intensity of all the bands, Soret and Q; the location of the three bands was preserved, but the whole bands were enlarged, as can be observed from Figure 12.

**Figure 13:**
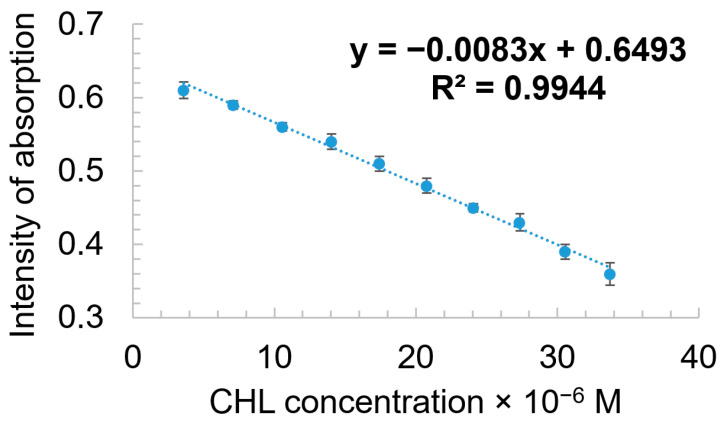
Linear dependence between the intensity of adsorption of AuNPs–TBAP complex and the CHL concentration in the range between 3.58 × 10^−6^ M and 3.37 × 10^−5^ M. Standard deviation ranges from 0.011 to 0.015.

The linear dependence between the intensity of the absorption of the AuNPs–TBAP complex read at 637 nm and the CHL concentration in the range between 3.58 × 10^−6^ M and 3.37 × 10^−5^ M is characterized by a very good coefficient of correlation (99.44%), as presented in Figure 13.

As can be concluded from the two experiments, the first one using the solely acidified TBAP–azaporphyrin and the second one being based on the same porphyrin synergistically complexed with AuNPs, these two methods are complementary, extending the detection of chloramphenicol from 3.58 × 10^−6^ M to 3.37 × 10^−5^ M, a domain that represents the relevant field for detecting the CHL levels in the human body fluids [65,66] and food [67,68,69], and in the industries where one must monitor for environmental control [70].

The sensitivity of the CHL detection method, that is the intensity of absorbance per concentration of chloramphenicol, is given by the slope of the fitting curve [71].

The limit of detection (LOD) was calculated for both the CHL and Co^2+^ detections using Equation (1).

LOD = 3.3 × N/B(1)

where N is standard deviation of the peak areas of the chloramphenicol or Co^2+^ and B is the slope of the corresponding calibration curve [72].

The value of LOD for CHL detection using the acidified AuNPs–TBAP complex, calculated by using Equation (1), is 0.98 × 10^−6^ M.

The intra-assay and inter-assay levels found for CHL security levels in milk (2.8–11.6 g kg^−1^), poultry (5–10.5 g kg^−1^), honey (4.7–5.6 g kg^−1^), and prawn (5.5–8.8 g kg^−1^) are highly recommended; we advise the use of our acidified AuNPs–azaporphyrin-based optical sensor for these screenings, because this sensor is covering these interest domains (values around 10^−5^ M) [73].

The CHL content in the blood for the “toxic” group was 2.8 µg/mL; this represents a concentration of 8 mM, meaning that our sensor can quantify the CHL levels in blood, a decade before becoming dangerous. The level of CHL concentration detected in urine that is putting human bodies in danger is 10–20 µg /mL, representing 6.19 × 10^−5^ M; this overlaps with the detection domain of our sensor, which is based on acidified AuNPs–azaporphyrin [74].

#### 3.5.4. Presumptions for the Mechanism of Detection of CHL Using a Plasmonic Complex Formed between Acidified TBAP and AuNPs

**Figure 14:**
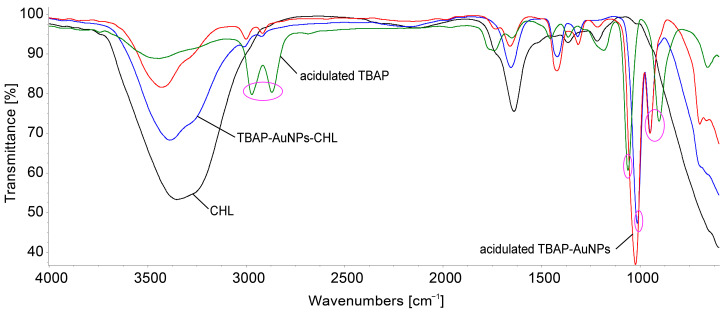
Comparison of the FT-IR spectra of all the involved compounds: the acidulated TBAP azaporphyrin, the complex between the AuNPs–TBAP, the complex AuNPs–TBAP after treatment with chloramphenicol TBAP–AuNPs-CHL, and the CHL alone. The differences between the Ft-IR spectra, marked in purple circles, are discussed in the text below.

Speaking about the mechanism of detection, we performed a comparison of the FT-IR spectra (Figure 14) for all the involved compounds: the acidulated TBAP azaporphyrin, the complex between the AuNPs–TBAP, the complex after treatment with chloramphenicol AuNPs–TBAP-CHL, and the CHL alone. These comparisons provided evidence of some important differences, which are highlighted by circles in Figure 14.

The aliphatic C-H stretching vibrations from tert-butyl groups around 2950–2860 cm^−1^ are very visible and obvious in the spectrum of acidified TBAP; these shift towards blue and are less intense in the complex with gold and in the AuNPs–TBAP-CHL due to the intake of water from the gold colloid. This phenomenon of shifting and reduction in the C-H bands is explained in the literature due to intermolecular hydrogen bonding [75,76]. The source of the broad absorption band at 3000–3500 cm^−1^ is the O-H stretching in the water molecules, as well as H-bonds and N-H stretching.

The band at around 1060 cm^−1^ from azaporphyrin, attributed to C-N vibrations in the ring, shifted to 1000 cm^−1^ in the AuNPs–TBAP complex after treatment with chloramphenicol; this means that there is an extended vibration to the C-N meso-C bonds that is caused by external interactions. On the other hand, the shifting of the IR peaks to lower wavenumbers is caused by the increased resonance that delocalize the electrons over more atoms than the two that were implied in the C-N bond, thus weakening this bond [77].

**Figure 15:**
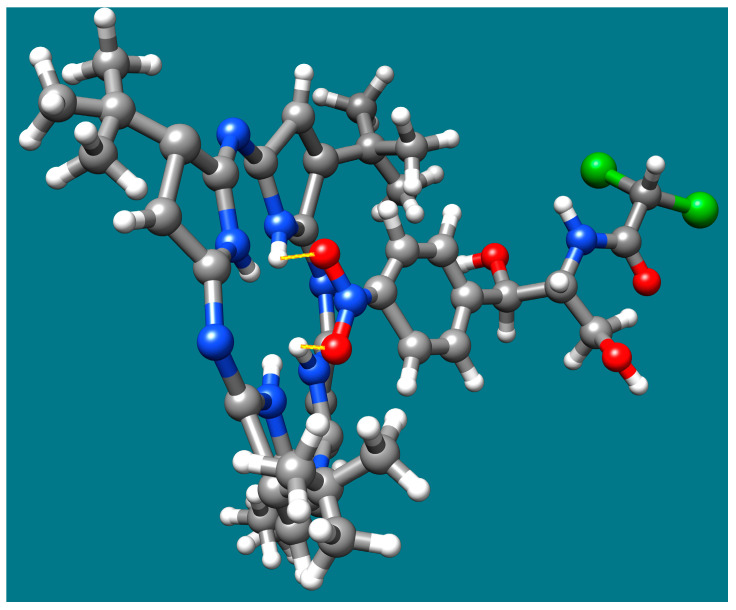
The detection mechanism, caused by electronic interactions between CHL and protonated azaporphyrin. Color code for atoms: hydrogen—white; carbon—grey; nitrogen—blue; oxygen—red; chlorine—green.

The band at around 900 cm^−1^ that is attributed to acidulated azaporphyrin, and which is assigned to the cycle of out-of-plane deformation, shifted to 960 cm^−1^ in the TBAP-AuNPs complex after treatment with chloramphenicol; this suggests that the bending of the C-C groups is taking place [6] and that the porphyrin is no more planar after interaction with CHL. The reduction in symmetry can generate the coupling of in-plane and out-of-plane vibrations; those around 960–980 cm^−1^ provide proof for the distortion of the macrocycle [78]. This distortion of the azaporphyrin might offer a suitable place for electronic interactions with CHL, as illustrated in Figure 15. These interactions take place between positively charged hydrogens linked to internal nitrogen atoms from the azaporphyrin core and the negatively charged oxygen atoms from CHL.

#### 3.5.5. Effect of Potentially Interfering Species in the CHL Detection

The influence of species that can interfere in the detection of chloramphenicol—such as niacin, lactic acid (LA), calcium gluconates (CaGlu), potassium iodide (KI), barium chloride (BaCl_2_), β-carotene, and ferric chloride (FeCl_3_)—was evaluated in the presence of chloramphenicol, using 100 times more concentrated solutions than that of CHL.

To the 3 mL AuNPs–TBAP complex solution in DMSO–water with CHL (c = 3.54 × 10^−6^ M), 0.1 mL solutions of anions and cations were added, which can interfere during the detection of chloramphenicol at a concentration of 1 × 10^−3^ M. Each sample was stirred for 1 min and the UV–Vis spectra were recorded.

The average percentage errors for the detection of chloramphenicol [32] is calculated according with Equation (2).

|ΔI|/I × 100(2)

where I is the absorbance intensity of the sample containing CHL and |ΔI| is the difference between the absorbance intensity of chloramphenicol and each of the studied interfering species, expressed in absolute value [79].

**Figure 16:**
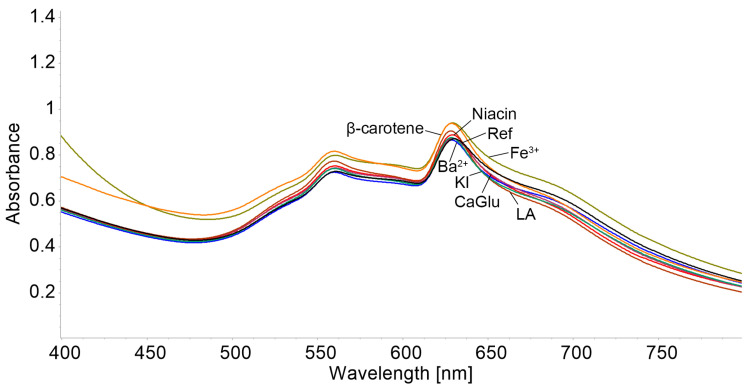
Overlaping UV–Vis spectra for the tested interfering compounds in the optical detection of CHL with AuNPs–TBAP complex.

**Figure 17:**
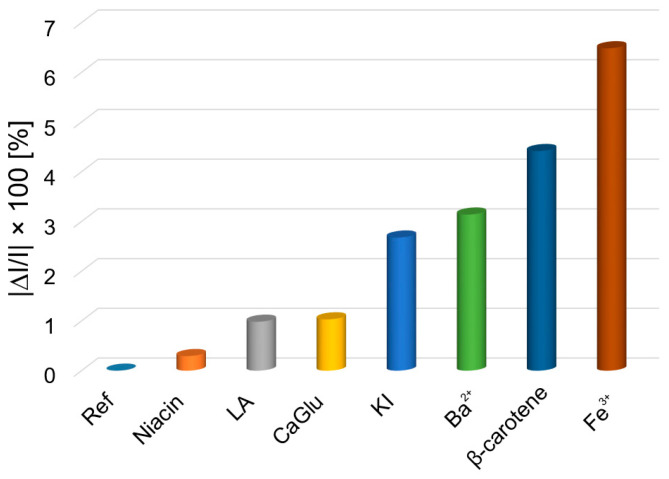
The graphical representation of the average percentage errors produced by interfering ions (concentrations are 100-fold higher) in the detection of CHL.

From Figure 16 and Figure 17, it can be concluded that the only significant interference is produced by FeCl_3_, introducing a percentage deviation of 6.49.

### 3.6. Detection of Co(II) from Aqueous Media Using the AuNPs–TBAP Complex, Generated in Water–DMSO Solution

Our first approach was to realize Co^2+^ detection by using acidified TBAP azaporphyrin alone. This study is presented in the Supplementary Materials and in Figures S5–S7.

#### 3.6.1. AuNPs–TBAP Complex Formation in Water–DMSO

The synergistic optical behavior of the hybrid materials formed between porphyrins and AuNPs have been previously analyzed and reported [62,63,80,81]; thus, we tried to obtain a new complex between the azaporphyrn and AuNPs in water–DMSO solution.

**Figure 18:**
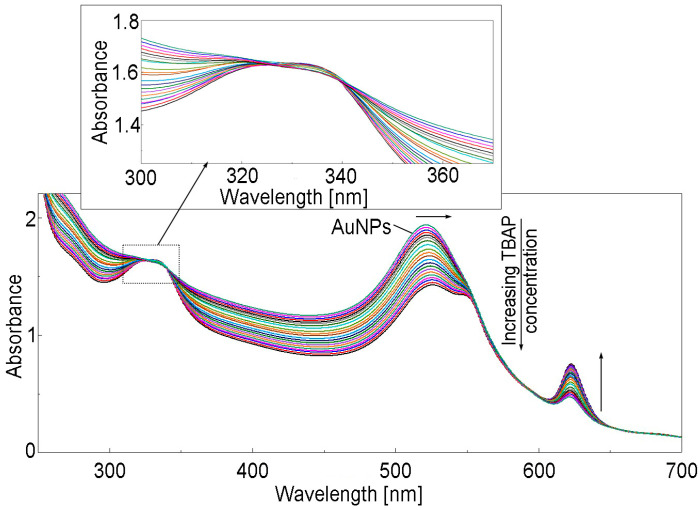
UV–Vis spectra of AuNPs–TBAP complex formation in the water–DMSO system.

Method for obtaining AuNPs–TBAP complex:

To 5 mL AuNPs in water (c = 6.918 × 10^−4^ M), different portions of TBAP in DMSO solution (c = 5.12406 × 10^−5^ M) were added. After every adding, the mixture was stirred for 90 s and the UV–Vis spectra were recorded. As can be observed in Figure 18, the plasmonic band of AuNPs is red-shifted from 518 nm to 525 nm; this occurs simultaneously with the decreasing intensity of the bands. A new peak shows up at 623 nm.

**Figure 19:**
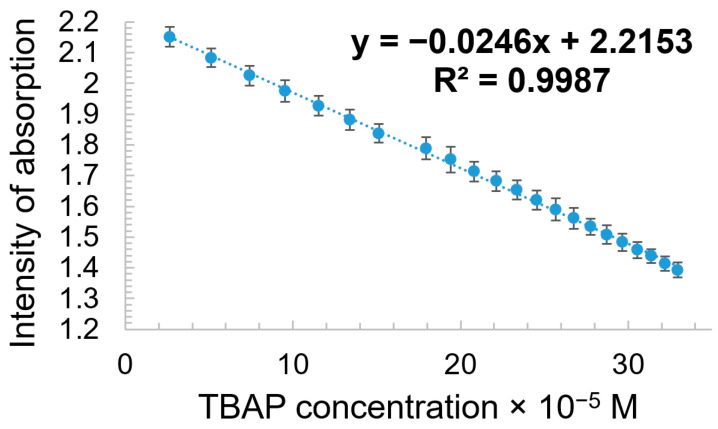
Linear dependence between intensity of absorption of AuNPs plasmon (read at 518 nm) and TBAP concentration in the range from 2.6 × 10^−5^ M to 3.3 × 10^−4^ M. Standard deviation ranges from 0.032 to 0.025.

As can be seen from the linear dependence shown in Figure 19, the azaporphyrin can be recognized and detected by AuNPs plasmon in a fast and simple way.

The linear dependence between the intensity of absorption of AuNPs plasmon (this time, read the newly formed peak at 623 nm) and TBAP concentration is presented in the Supplementary Materials, Figure S7, in the same concentration interval from 2.66 × 10^−5^ M to 3.29 × 10^−4^ M; this shows that, in this simple way, the azaporphyrin can be quantified after being used in the imaging of cancer cells [82,83,84,85,86,87].

#### 3.6.2. UV–Vis Detection of Co^2+^ Using AuNPs–TBAP Complex

The spectrophotometric detection of Co^2+^ ions was performed using the complex obtained between AuNPs and TBAP in water–DMSO.

**Figure 20:**
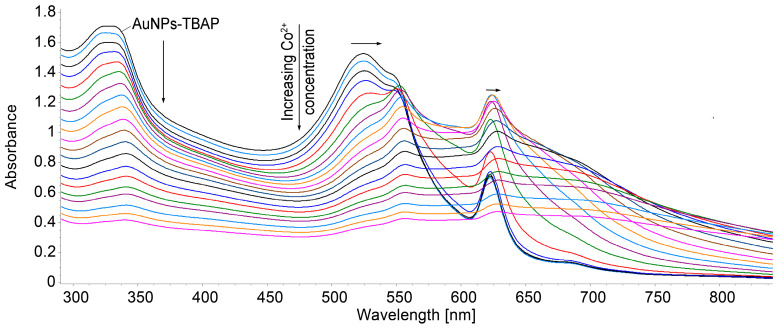
Overlapping UV–Vis spectra of AuNPs- TBAP complex in water–DMSO after stepwise addition of Co^2+^.

**Figure 21:**
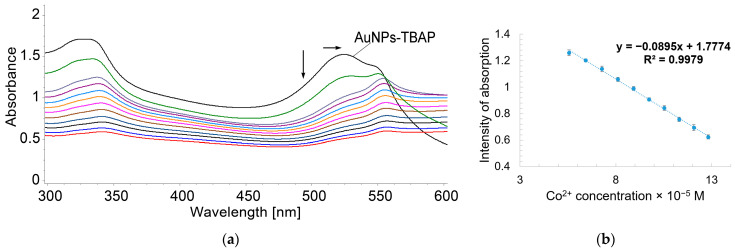
(**a**) Selected UV–Vis spectra of AuNPs–TBAP complex in water–DMSO after stepwise addition of Co^2+^; (**b**) linear dependence between intensity of absorption of AuNPs–TBAP in water–DMSO read at 555 nm and Co^2+^ concentration (5.55 × 10^−5^ to 1.28 × 10^−4^ M). Standard deviation ranges from 0.02 to 0.016.

To 5 mL solutions of AuNPs–TBAP, different portions of cobalt acetate in water were added (c = 1 × 10^−3^ M). The obtained mixtures were stirred for 1 min at room temperature, and the UV–Vis spectra were recorded (Figure 20). A selection of the UV–Vis spectra of AuNPs–TBAP complex in water–DMSO during the increasing concentrations of Co^2+^, offering fitted values for linear dependence, is shown in Figure 21.

The value of the LOD for Co^2+^ detection when using the AuNPs–TBAP complex, calculated using Equation (1), is 1.3 × 10^−5^.

Despite the fact that cobalt has been reported to have a low oral toxicity, reports regarding cardiomyopathy [88], due to excessive cobalt intake, have asked for its strict monitoring in different foods.

With respect to Co^2+^ analysis, our sensor might be appropriate for fast and accurate detection of its content in the following seeds, spices, vegetables, milk, and meat products: instant coffee, lamb kidney, Brazil nuts, chocolate milk, linseeds, brewers’ yeast, millet seeds, buckwheat, kidney beans, chocolate, dark, rice, chili powder, sunflower kernels, liver, bovine, curry powder, cashew nuts, peanut butter, potato, fresh, broccoli, and brown lentils.

#### 3.6.3. Potential Mechanism of Co^2+^ Detection Based on Compared FT-IR Spectra of AuNPs–TBAP Complex and AuNPs–TBAP-Co^2+^

**Figure 22:**
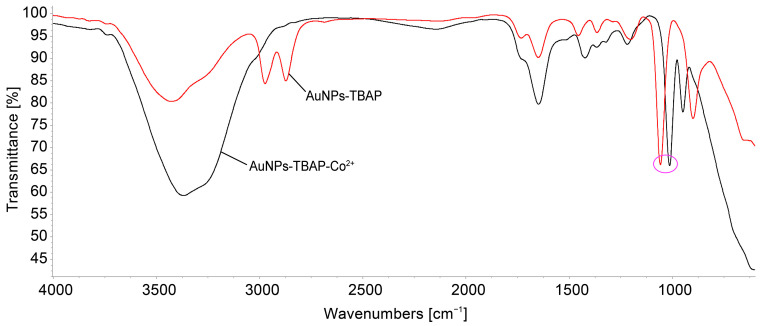
Comparison of IR spectra for the sensitive material before and after Co^2+^ detection. The circle represents the shifting of the band from 1057 cm^−1^ to the lower wavenumbers (1010 cm^−1)^ assigned to the newly coordinated bond between Co^2+^ and the internal nitrogen atoms of azaporphyrin.

Figure 22 represents FT-IR spectra of the AuNPs–TBAP complex and the AuNPs–TBAP complex after being exposed to Co^2+^. The band situated around 1057 cm^−1^ that belongs to the AuNPs–TBAP complex is shifted to lower wavenumbers (1010 cm^−1^) after the complex is treated with Co^2+^, thus revealing the formation of a potential coordinative bond formation between Co^2+^ and the internal nitrogen atoms. As a consequence, the bond strength has increased. This band is reported [89] to be metal-sensitive and to contain significant Co-N stretching and N-Co-N in-plane bending vibrations.

**Figure 23:**
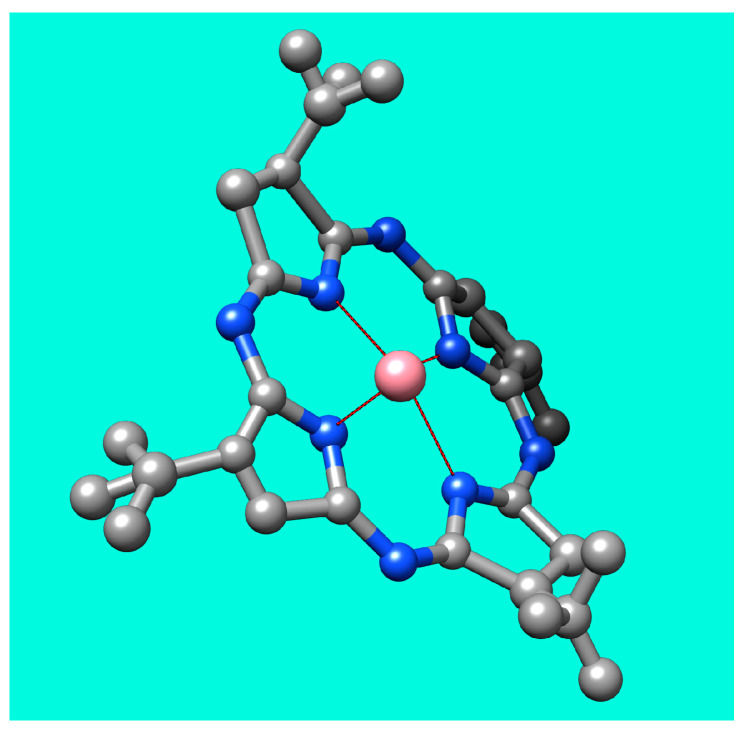
The detection mechanism cause by electronic interactions between Co^2+^ and azaporphyrin. The color code for the atoms is as follows: carbon—grey; nitrogen—blue; Co^2+^—pink.

The proposed mechanism for Co^2+^ recognition is presented in Figure 23. 

#### 3.6.4. Effect of Interfering Species for the UV–Vis Detection of Co^2+^

The reference sample is comprised of 3 mL AuNPs–TBAP complex plus 0.1 mL Co^2+^ solution, in which 0.1 mL bi-distilled water was added. In each of the other samples, 0.1 mL solution of each interfering compound was added instead of 0.1 mL water in order to avoid false results due to dilution when measuring the interferences effects. The appropriately selected interfering compounds, with concentrations of 10^−2^ M, are as follows: NaCl (natrium chloride), CaL (calcium lactate), KCl (potassium chloride), Glu (glucose), MnCl_2_ (manganese chloride), and KI (potassium iodide). Every mixture was stirred for 90 s and the UV–Vis spectra were recorded. Each added interfering compound lead to a 100-fold higher concentration in the solution when compared to that of Co^2+^ acetate.

The interference species that were chosen are frequently found in human serum and water environments; therefore, they might influence the detection of the Co^2+^ ions. In our case, the only significant effect that was found to be disturbing the accurate measurement was produced by KI. The rest of the selected species generated interference effects that were lower than 5%.

**Figure 24:**
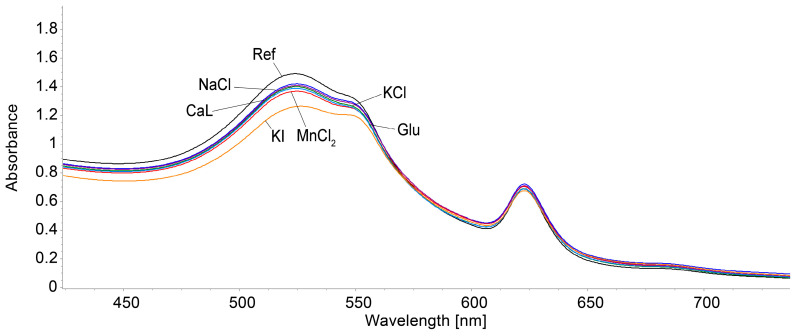
Overlapping UV–Vis spectra showing the effects generated by the tested interfering compounds: NaCl (natrium chloride), CaL (calcium lactate), KCl, Glu (glucose), MnCl_2_, and KI during the optical detection of Co^2+^ using the AuNPs–TBAP complex.

**Figure 25:**
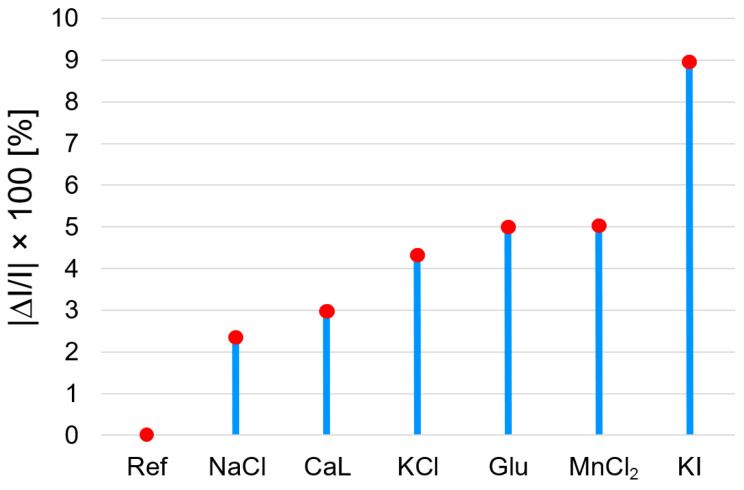
The graphical representation of the average percentage errors produced by interfering species: NaCl, CaL (calcium lactate), KCl, Glu (glucose), MnCl_2_, and KI (absorption read 555 nm) in the detection of Co^2+^.

From Figure 24 and Figure 25, it can be concluded that the only significant interference is produced by KI, with a percentage deviation of 9; this means that this method cannot be applied for thyroid-dysfunctional people.

The average percentage errors for the detection of Co^2+^ ions are calculated with Equation (2).

## 4. Conclusions

The purpose of this work was to develop two new optical sensors for the detection of an antibiotic (chloramphenicol) and of cobalt from water environment. This was performed using the same porphyrin or its plasmonic hybrid materials, made with AuNPs. The TBAP showed multifunctionality and optical capabilities, both on its own and as a synergic component for hybrid materials obtained with AuNPs, both in acid and in organic media. The results attained in the detection of CHL, using an acidified TBAP–AuNPs complex, showed a linear correlation between the intensity of the sensitive material and CHL concentrations in a range between 3.58 × 10^−6^ M and 3.37 × 10^−5^ M. For the Co^2+^ ions, the detection was also linear in a range from 5.55 × 10^−5^ to 1.28 × 10^−4^ M using the AuNPs–TBAP complex.

The detection mechanism of CHL is based on the electronic interactions that take place in acid media between positively charged hydrogens linked to internal nitrogen atoms from azaporphyrin core and the negatively charged oxygen atoms from CHL. In addition, the distortion of TBAP–azaporphyrin might offer a suitable place for electronic interactions with CHL to occur.

The detection mechanism of Co^2+^ can be explained based on the new IR band formed around 1010 cm^−1^ after the AuNPs–TBAP complex is treated with Co^2+^, revealing the formation of coordinative bonds (Co-N stretching and N-Co-N in-plane bending vibrations [89]) between Co^2+^ and the internal nitrogen atoms from TBAP azaporphyrin.

Taking the detection domain of our best formulated sensors into consideration, they might be appropriate for quantifying the CHL levels in blood a decade before this antibiotic becomes dangerous; this could also be performed in urine, where the concentration that is putting human bodies in danger overlaps with the detection domain of our sensor based on acidified AuNPs–azaporphyrin [74].

## Data Availability

The data are available by request from authors.

## Supplementary Materials

The following supporting information can be downloaded at: https://www.mdpi.com/article/10.3390/biomedicines12040770/s1. Figure S1: UV–Vis spectra of TBAP solutions in DMSO, with different concentrations. Determining the concentration level that evidences the formation of aggregates (c = 1.15 × 10^−4^ M, where the splitting is taking place). Figure S2: Overlapping UV–Vis spectra of TBAP in DMSO upon increasing acidity from pH = 9.5 to pH = 1.77 by addition of HCl (c = 37%). Figure S3: UV–Vis spectra of TBAP at basic pH in DMSO/water. The blue-shifted and split Soret band and generation of only one Q band. Figure S4: The color of a solution of TBAP in DMSO (c = 4.6 × 10^−5^ M) under illuminating at UV–Vis lamp at different selected wavelengths. Figure S5: Overlapping spectra of acidified TBAP solution in DMSO after stepwise addition of Co^2+^ in a large range of concentrations, 9.70 × 10^−6^ M−1.77 × 10^−4^ M. Figure S6: Linear dependence between intensity of absorption of acidified TBAP read at 335.5 nm and Co^2+^ concentration, validated in a range of Co^2+^ concentrations: 8.92 × 10^−5^ M–1.77 × 10^−4^ M. Figure S7: Linear dependence between intensity of absorption of AuNPs read at 623 nm and TBAP concentration. Table S1: The most efficient and recently used methods for chloramphenicol detection. Table S2: Various methods for Co^2+^ detection from different samples. Detection domains, detection limits, and areas of application. References [17,18,23,24,25,26,27,28,58,77,90,91,92,93,94,95,96,97,98,99,100,101,102,103,104,105,106,107,108,109,110,111,112,113,114,115,116,117,118,119,120,121,122,123] are citied in the Supplementary materials.
