# Supplementary material for: Nanomaterials Based on 2,7,12,17-Tetra-tert-butyl-5,10,15,20-tetraaza-21H,23H-porphine Exhibiting Bifunctional Sensitivity for Monitoring Chloramphenicol and Co2+
Source: Biomedicines. 2024 Mar 30;12(4):770. doi: 10.3390/biomedicines12040770 (PMC11047853; doi:10.3390/biomedicines12040770)
Supplement: Supplementary file 1 [file biomedicines-12-00770-s001.zip › biomedicines-2913856-supplementary.pdf]

Nanomaterials Based on 2,7,12,17-Tetra-tert-butyl-5,10,15,20-tetraaza-21H,23H-porphine Exhibiting Bifunctional Sensitivity for Monitoring Chloramphenicol and Co^{2+}

Ionela Fringu [1], Diana Anghel [1], Ion Fratilescu [1], Camelia Epuran [1], Mihaela Birdeanu [2] and Eugenia Fagadar-Cosma [1,*]

1 Institute of Chemistry "Coriolan Dragulescu", Mihai Viteazu Avenue 24, 300223 Timisoara, Romania; mcreanga@acad-icht.tm.edu.ro (I.F.); danghel@acad-icht.tm.edu.ro (D.A.); ionfratilescu@acad-icht.tm.edu.ro (I.F.); ecamelia@acad-icht.tm.edu.ro (C.E.)
2 National Institute for Research and Development in Electrochemistry and Condensed Matter,
P. Andronescu Street, No. 1, 300224 Timisoara, Romania; mihaelabirdeanu@gmail.com
* Correspondence: efagadar@yahoo.com

Table S1 presents a situation regarding the most efficient and recently used methods for chloramphenicol detection, based on porphyrins or other materials.

Table S1. The most efficient and recently used methods for Chloramphenicol detection.

**Sensitive compound**		**Detection method**	**Detection domain Values from cited articles/ Values calculated in μM**	**Detection limit** **Values from cited articles/ Values calculated in μM**	**Ref.**
Containing porphyrins as sensitive compounds	Composite zirconium-porphyrin (tetrakis(4-carboxyphenyl)porphyrin - MOF-based	Fluorescent sensing	0.1 pg mL^{-1} – 10 ng mL^{-1} / 0.15 – 13.6 μM	0.08 pg mL^{-1} / 1.11 × 10^{-8} μM	[90]
	Composite zirconium octahedral clusters and TCPP (tetrakis (4-carboxyphenyl) porphyrin) ligand	Electrochemical detection by cyclic voltammetry and differential pulse voltammetry	0.01 – 0.80 μM	0.0018 μM	[17]
	Composite AuNPs - (Meso-tetra(carboxyphenyl)	Electrochemical aptasensor	-	0.0138 ng mL^{-1} / 0.022 μM	[91]

	porphyrin, Zirconium tetrachloride				
	Zirconium-porphyrin MOF in-situ synthesized with GO functionalized with –COOH group	Ultra-sensitive electrochemical	0.01 ng/mL to 50 ng/mL / 3×10^{-5} – 0.16 μM	7.04 pg/mL / 0.05 μM	[18]
Based on other sensitive compounds	Chlorine-functionalized graphene oxide (Cl-RGO)	Electrochemical sensor	2 – 35 μM	1 μM	[92]
	Palladium nanoparticles decorated graphene oxide nanocomposite	Electrochemical sensor	50 – 1000 nM / 0.05 – 1 μM	50 nM / 0.05 μM	[58]
	Eu_2O_3 nanoparticles decorated reduced graphene oxide	Amperometric sensor	0.02 – 800 μM	1.32 nM / 0.0013 μM	[93]
	Bimetallic core–shell nanoparticles consisting of a gold core, silver shell, and a mesoporous silica coating	Surface-enhanced Raman spectroscopy (SERS)	-	6.68×10^{-8} M / 0.0668 μM	[94]
Aptasensor based on modified glassy-carbon electrode rGO/AgNPs		Electrochemical detection	0.01 – 35 μM	2 nM / 0.002 μM	[95]
Acidified TBAP-AuNPs complex	CHL	Optical sensor	3.58×10^{-6} M – 3.37×10^{-5}	0.98×10^{-6}	This work

Table S2 presents some recent performances in Co^{2+} detection.

Table S2. Various methods for Co^{2+} detection from different samples. Detection domains, detection limits and areas of applications.

Sensitive compound		Detection method	Detection Domain Values from cited articles/ Values calculated in μM	Detection limit	Application	Ref.
				Values from cited articles/ Values calculated in μM		
A quinoline-based hydrazone, namely, bis((quinolin-8-yl)methylene)carbonohydrazide	Co^{2+}	Colorimetric sensor	-	0.21 μM	-	[96]
	Zn^{2+}	Fluorescent sensor	-	0.66 μM	Biological applications	
Green-fluorescent N-doped carbon dots -fabricated using hydrothermal treatment of tyrosine and urea	Co^{2+}	-	0.1 μM –10 μM; 25 μM – 275μM; 300 μM – 400μM	0.15 μM	Biomedical field - temperature sensor with a linear range of 25 – 80 °C	[97]
Thiosemicarbazone-linked acetylpyrazine	Co^{2+}	Colorimetric detection	-	1.637 μM	Environmental water samples	[98]
Ultrasonic assisted cloud point extraction method	Co^{2+}	UV–Vis spectrophotometer	0.0169 – 0.169 μM<br><br>0.1695 – 3.5593 μM	0.76 μg L^{-1}/ 0.012 μM	Determination of Cobalt (II) (Co(II)) in spices.	[99]
Dithizone used in chromogenic sensing of CN^- as an anionic species and for Co^{2+} as a cationic species in DMSO/H_2O media.	Co^{2+}	Colorimetric	0.3 – 4.4 μM	0.04 μM	Application in a variety of cases requiring rapid and accurate analysis of Co^{2+} and CN^- from human serum and water samples	[100]
	CN^-		3.3 – 58.6 μM	0.43 μM		
Carboxyl-functionalized CdS QDs	Co^{2+}	Colorimetric detection	8.47–237.3μM	0.23 μg mL^{-1}/ 3.89 μM	Environmental analysis	[101]

2,6,7-trihydroxy-9-(4A-chlorophenyl)-3-fluorone oxidized with hydrogen peroxide in alkaline solution	Co^{2+}	Chemiluminescence	-	0.07 ng mL^{-1}/ 0.175 μM	Water sample	[102]
Retention of cobalt ions with polyvinyl chloride modified by 3-(2-thiazolylazo)-2,6-diaminopyridine in a mini-column system.	Co^{2+}	Atomic absorption spectrometry	-	1.3 μgL^{-1}/ 0.027 μM	Sample waters, waste water, vegetable, fruit and drug with complicated matrices	[103]
A polyaniline/platinum (PANI/Pt) bilayer fiber optic sensor based on surface plasmon resonance phenomenon	Co^{2+}	Surface plasmon resonance (SPR)	-	0.14 μM	Environmental water quality control detection of cobalt ions (Co^{2+}) in an aqueous solution	[104]
Spectrofluorimetric determination due to complex formed with 8-hydroxyquinolein (8-HQ) in aqueous solution	Co^{2+}	Spectrofluorimetric determination	7.3×10^{-2} μg L^{-1} 4.12×10^{-3} μg L^{-1}/ 0.503 – 0.028 μM	2.19×10^{-2} μgL^{-1}/ 1.51×10^{-4} μM<br>4.3μ10^{-2} μg L^{-1}/ 2.96×10^{-4} μM	Detection from tap waters and foods samples (milk powder, express coffee, cocoa powder)	[105]
AgNPs-glutathione	Co^{2+}	Surface plasmon resonance (SPR)	-	0.68 μM	Highly sensitive and selective in aqueous systems for detection of cobalt ions (Co(II))	[106]
Polyvinyl chloride incorporating 5-[o-carboxyphenylazo]2,4-	Co^{2+}	Optical sensor	0.05 – 45.20 μM	0.015 μM	Environmental samples	[107]

dihydroxybenzoic acid and sodium tetraphenylborate, plasticized with dioctyl adipate						
1,5-bis(di-2-pyridyl) methylene thiocarbohy-drazide (DPTH) was used as a chelating agent	Co^{2+}	Flame atomic absorption spectrometry	-	12.4 ng mL^{-1}/ 0.21 μM	Method for the determination of cobalt from food, environmental and water samples	[108]
The procedure is based on the formation of the hydrophobic complex between Co(II) and 4-methoxy-2-sulfo-benzenediazoaminoazo-benzene	Co^{2+}	Flame atomic absorption spectrometry (FAAS)	-	0.47 ng mL^{-1}/ 0.0079 μM	Trace amounts of cobalt from water and food samples	[109]
Choline chloride and phenol in a 1:2 M ratio was used as a green solvent to extract cobalt from the aqueous sample solution.	Co^{2+}	Flame atomic absorption spectrometry (FAAS)	5.0 – 50 μg L^{-1}/ 0.084 – 0.84 μM	2μg L^{-1}/ 0.033 μM	Detection from linden tea samples	[110]
N-(2-(2-butyl-1,3-dioxo-2,3-dihydro-1H-benzo[de]isoquinolin-6-yl)hydrazine-1-carbonothioyl)benzamide	Co^{2+}	Fluorescent method	-	0.26 μM	Highly selective fluorescent chemosensor for biological environments	[111]
A Chrysoidine G (CG) chemosensor was exposed to cobalt	Co^{2+}	Colorimetric *in situ* method	0.4 –1 ppm / 0.14 – 0.35 μM	2 ppm/ 0.07 μM	Practical applications such as on-site test kits and real-time monitoring	[112]
A biomass-derived adsorbent	Co^{2+}	Dispersive solid-phase extraction	-	1.9 μg L^{-1}/ 0.032 μM	Food samples	[113]

2-(5-Bromo-2-pyridylazo)-5-[N-n-propyl-N-(3-sulfopropyl) amino]	Co^{2+}	Co (II) liquid ion-selective electrode	3.4 × 10⁻⁸ – 2.4 × 10⁻² M / 0.034 – 24000 µM	2.7 ×10⁻⁸ M / 0.027µM	Food products and pharmaceutical samples	[114]
1-nitroso-2-naphthol	Co^{2+}	Liquid phase microextraction), with subsequent detection by digital image colorimetry	-	0.8 µg L⁻¹/ 0.013 µM	Food, dietary supplement and water samples	[115]
AuNPs-TBAP complex	Co^{2+}	Optical sensor	5.55 × 10⁻⁵ to 1.28 × 10⁻⁴ M	1.3 × 10⁻⁵ M	milk and meat products: coffee instant, lamb kidney, Brazil nuts	This work

1. Aggregation Tests Monitored in UV-Vis for TBAP in DMSO Solutions at Different Concentrations

During this study it was important to establish the maximum concentration that provide non-aggregated species for TBAP in DMSO solutions [119].

To achieve this, solutions of TBAP of different concentrations were prepared in DMSO as follows: 1.15 × 10^{-3} M; 2.3 × 10^{-4} M; 4.6 × 10^{-5} M; 9.2 × 10^{-6} M; 1.84 ×10^{-8} M.

The porphyrin solutions with concentrations lower than 1.15 × 10^{-4} M are preserving their monomeric state in DMSO solution (Figure S1). As a result of aggregation process, at higher concentrations than 1.15 × 10^{-4} M, the TBAP azaporphyrin displays in UV-Vis the split of the Soret band, confirming the generation of *J*-type aggregates which show a red-shifted Soret branch to 350 nm and of *H*-type aggregates that exhibit a blue-shifted branch to 312 nm.

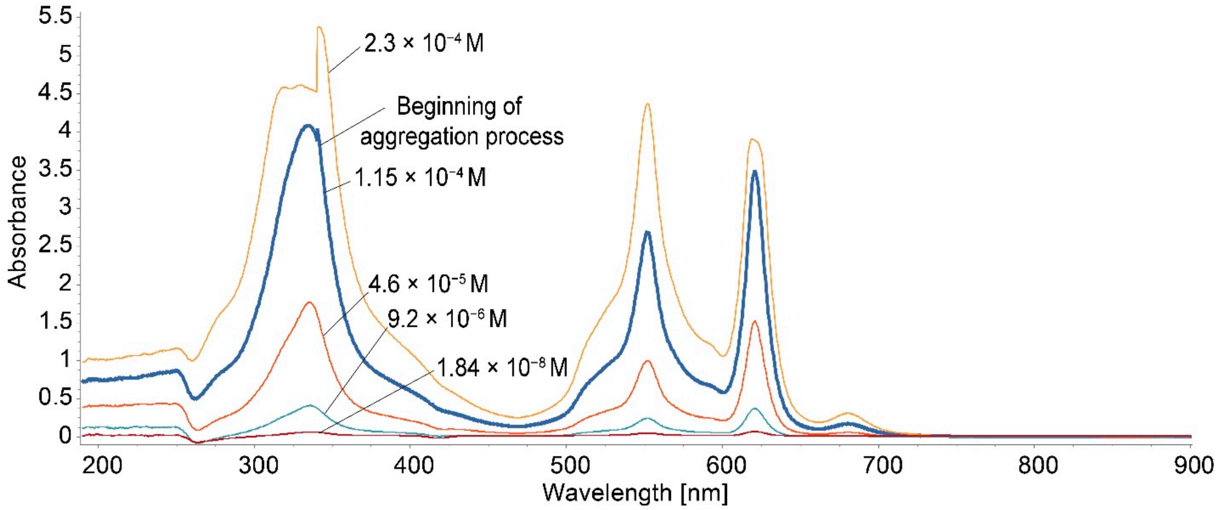

Figure S1. UV-Vis spectra of TBAP solutions in DMSO having different concentrations. Determining the concentration level that is evidencing the formation of aggregates (c = 1.15 × 10^{-4} M)

2. The UV-Vis Optical Behavior of TBAP in DMSO in Acid-Base Tests

2.1. Behavior of TBAP in Acid Medium Monitored by UV-Vis Spectroscopy

To 5 mL TBAP solution in DMSO (c = 6.691 × 10^{-5} M), small portions between 0.01-0.5 mL of HCl (c= 37%) were added and the UV-Vis spectra were recorded and presented in Figure S2. As resulted from Figure S2, the allure of the spectra is maintained fairly unchanged, the position of the bands suffers very-small shifting to the red, but due to dilution phenomena the intensity of the bands decreased continuously by increasing the acid quantity. Therefore, it can be stated that in acid media the TBAP azaporphyrin is preserving intact its shape and only the absorption intensity is depending of acid concentration levels.

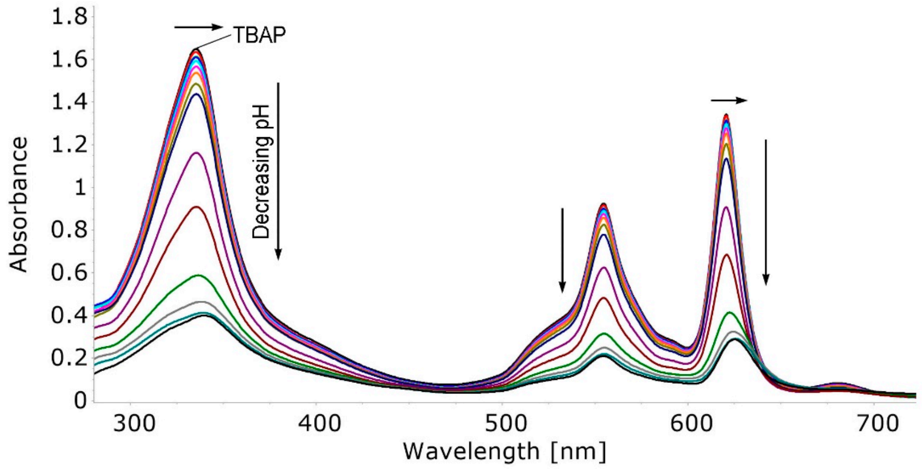

Figure S2. Overlapped UV-Vis spectra of TBAP in DMSO upon increasing acidity from pH= 9.5 to pH= 1.77 by addition of HCl (c= 37%).

2.2. The Behavior of TBAP in Base Medium Tested by UV-Vis Spectroscopy

To a solution consisting of 5 mL TBAP in DMSO (c= 6.691 × 10^{-5} M), portions of 1M NaOH solution of 0.01 mL are added continuously. The changing of color from dark blue to turquoise is immediately seen, after each base solution is added. This change is reversible after 5-10 minutes, if no more NaOH solution is added.

In Figure S3, the changes regarding the shape of the UV-*V*is spectra at different basic pH associated with modification of the color are observed. After adding of 0.07 mL of 1M NaOH solution the pH reaches the value of 14 and the color is turquoise for a longer time (due to formation of the dianions in the presence of strong bases in *non-aqueous* media [117,118]).

After the solution is reaching a pH of 8.7, some important changes regarding optical behavior are occurring. The first important change is the enlargement of Soret band and its splitting into a blue shifted one, associated with H-type aggregation and a significant red shifted one (bathochromic shift of 60 nm), assigned to J-type aggregation. The second important change is that the two Q_x and Q_y bands are unified into one Q band (Figure S3) that suffer hyperchromic modification, and becomes the highest intensity band of the spectrum. This Q band located in the visible range and possessing a high molar extinction coefficient of 4.45, might be exploited for photodynamic therapy (PDT) of different types of cancer offering both suitable energy and deep penetration for ill cells without harming the healthy cells [119-121].

At higher pH values, between 11.5 to 13.7, as displayed in Figure S3, until reaching the pH = 14, it can be seen the continuously increase in intensity of Q_x band with significant hipsochromic shift and a major decrease of Q_y band. At pH 14, the UV-Vis of azaporphyrin displays only one distinctive Q band situated around 610 nm.

This behavior is a consequence of the fact that the porphyrazine ring can lead to ring contraction [122] because nitrogen atoms can participate both in the redistribution of electron density over σ-bonds and in the π-π conjugation of the entire macrocycle. The tert-butyl substituents together with the eight nitrogen

atoms present in the macrocycle might determine a decrease in the strength of the NH-bond and thus influence the formation and stability of the anion species [123]. The distortion of molecular conformation leads also to aggregation-induced enhanced emission [77] as illustrated in Figure S4 for fuchsia colored sample.

Regarding dianionic species, significant saddle-type distortion was observed accompanied by an increase with 0.11 Å of the diameter of the N4 internal core, so that the interactions with metals and molecules are easier [118]. The same report also revealed that in the case of β-alkyl substitution of azaporphyrins, it might appear a small decrease regarding the capacity for performing the first deprotonation, but the stability of the dianions is greater.

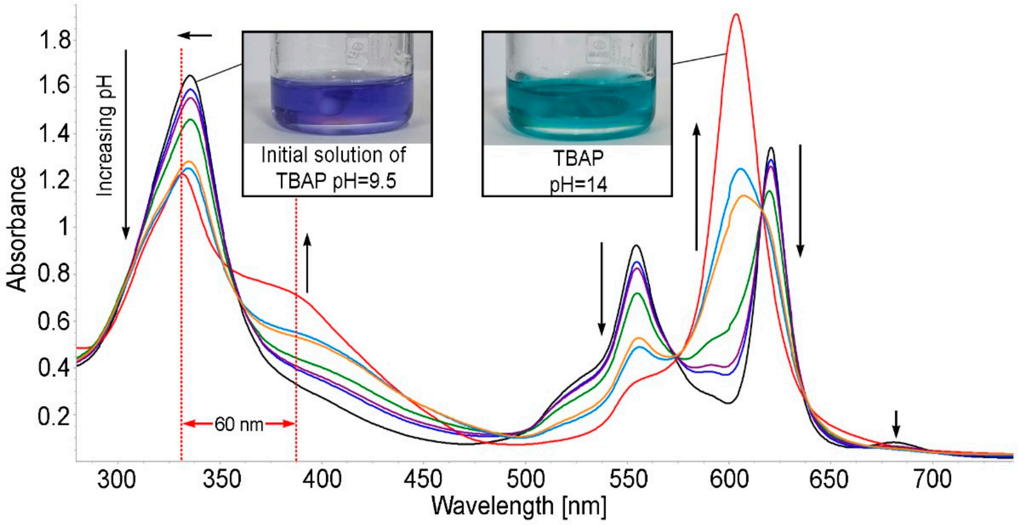

Figure S3. UV-vis spectra of TBAP at basic pH in DMSO/water. The blue-shifted and splitted Soret band and generation of only one Q band.

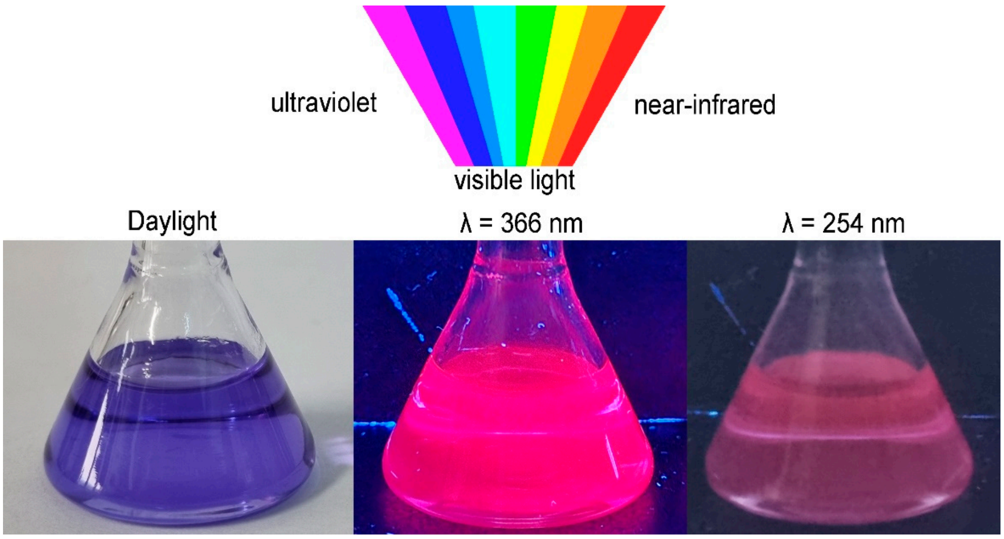

Figure S4. The color of a solution of TBAP in DMSO (c= 4.6 × 10^{-5} M) under illuminating at UV-Vis lamp at different selected wavelengths.

Optical detection of Co^{2+} using acidified TBAP azaporphyrin alone

Our first approach was to realize the Co^{2+} detection by using acidified TBAP azaporphyrin alone. The UV-vis spectra of acidified TBAP solution in DMSO after stepwise addition of Co^{2+} are presented in Figure S5.

Method for detection Co^{2+} ions

To a quantity of 5 mL TBAP solution in DMSO (c = 5.124 × 10^{-5} M) acidified with 0.1 mL HCl (37 %), to reach pH=1.77, portions of 0.05 mL or 0.1 mL of cobalt acetate, $Co(OAc)_2$, in water (c= 1 × 10^{-3} M) were added. The mixture was stirred for 90 sec and UV-Vis spectra were recorded. The isosbestic point that appears at 634 nm, associated with small red shifted Qx band indicates the interactions between Co and azaporphyrin (Figure S5).

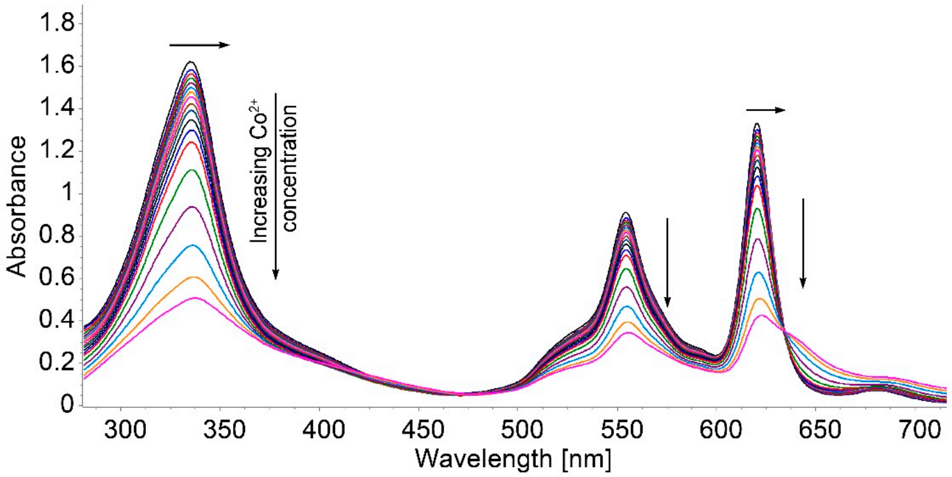

Figure S5. Overlapped spectra of acidified TBAP solution in DMSO after stepwise addition of Co^{2+} in a large range of concentrations 9.70 × 10^{-6} M – 1.77 × 10^{-4} M.

The dependence between the intensity of absorption of acidified TBAP read at 335.5 nm and Co^{2+} concentration, is presented in Figure S6 and is linear in the concentration interval 8.92 × 10^{-5} M – 1.77 × 10^{-4} M which is relevant for the recognition of Co^{2+} ions from different water environment and foods, such as: millet samples and water, tap water, mineral water, orange juice and beer [23,24].

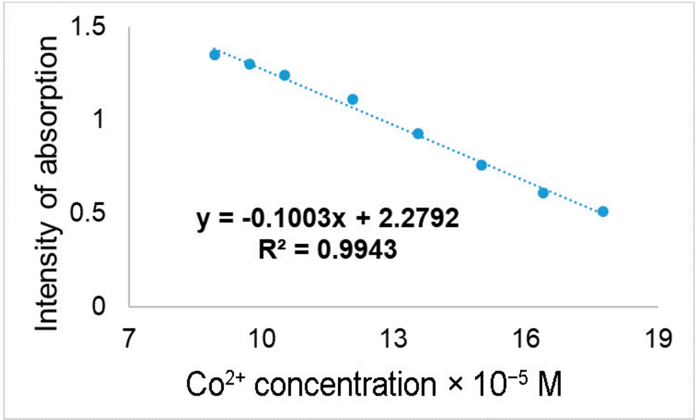

Figure S6. Linear dependence between intensity of absorption of acidified TBAP read at 335.5 nm and Co^{2+} concentration, validated in the range of Co^{2+} concentrations 8.92 × 10^{-5} M – 1.77 × 10^{-4} M.

Method for obtaining the complex between TBAP and AuNPs in water/DMSO solution

In this study the ability of AuNPs to be used for the detection of azaporphyrin was demonstrated. This is a new tool for monitoring porphyrin retention after cancer imaging procedures or for porphyria disease diagnosis [25]. The synergistic optical behaviour of the hybrid plasmonic materials formed between porphyrins and AuNPs have been previously analyzed and reported [26-28].

Azaporphyrin can be accurately monitored (Figure S7) in the concentration range from 2.66 × 10^{-5} M to 3.29 × 10^{-4} M) by reading the intensity values on the wide plasmon at 623 nm, formed during the addition of azaporphyrin to the sensitive AuNPs, according to Figure 18 from the main text.

.

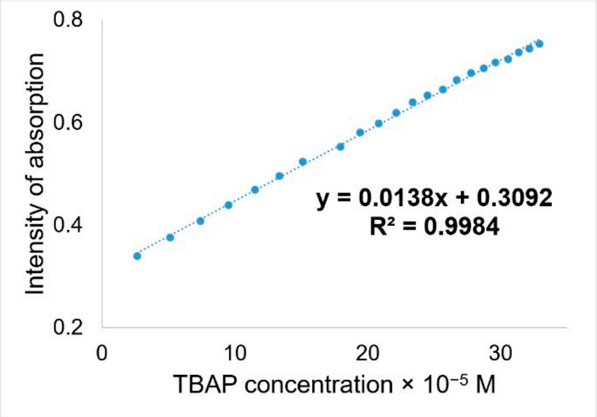

Figure S7. Linear dependence between intensity of absorption of AuNPs read at 623 nm and TBAP concentration.

References

17. Wei, C.; Zhou, H.; Liu, Q. PCN-222 MOF decorated conductive PEDOT films for sensitive electrochemical determination of chloramphenicol. *Mater. Chem. Phys.* **2021**, *270*, 124831. https://doi.org/10.1016/j.matchemphys.2021.124831.

18. Li, H.K.; Ye, H.L.; Zhao, X.X.; Sun, X.L.; Zhu, Q.Q.; Han, Z.Y.; Yuan, R.; He, H. Artful union of a zirconium-porphyrin MOF/GO composite for fabricating an aptamer-based electrochemical sensor with superb detecting performance. *Chin. Chem. Lett.* **2021**, *32*, 2851–2855. https://doi.org/10.1016/j.cclet.2021.02.042.

23. Paleologos, E.K.; Prodromidis, M.I.; Giokas, D.L.; Pappas, A.C.; Karayannis, M.I. Highly selective spectrophotometric determination of trace cobalt and development of a reagentless fiber-optic sensor. *Anal. Chim. Acta* **2002**, *467*, 205–215. https://doi.org/10.1016/S0003-2670(02)00094-6.

24. Jiang, Z.-T.; Li, R.; Zhang, J.-C. Determination of cobalt in foods using β-cyclodextrin epichlorohydrin polymer functionalized with 1-(2-pyridylazo)-2-naphthol. *JFDA* **2004**, *12*, 7. https://doi.org/10.38212/2224-6614.2651.

25. Zhang, R.; Zhong, Y.; Hu, Y.; Chen, Y.; Xia, L.; Li, G. Liquid-Phase Cyclic Chemiluminescence for the Identification of Cobalt Speciation. *Anal. Chem.* **2024**, *96*, 3933–3941. https://doi.org/10.1021/acs.analchem.3c05864.

26. Genchi, G.; Genchi, G.; Catalano, A.; Carocci, A.; Sinicropi, M.S. Prevalence of Cobalt in the Environment and Its Role in Biological Processes. *Biology* **2023**, *12*, 1335. https://doi.org/10.3390/biology12101335.

27. Saulea, M.; Stoica, A.I., Baiulescu, G.E.; Marinescu, D.; Ionica, M. Determination of cobalt in food samples. *Rev. Chim.* **2004**, *55*, 301–303.

28. López, O.N.B.; Fuentes, H.C.; Perezgasga, F.V.; Casillas, H.A.M.; Chamarro, J.A. Detection and analysis of cobalt in continuous flow using an analytical microsystem based on LTCC technology. *Sens. Actuators B Chem*. **2016**, *227*, 11–16. https://doi.org/10.1016/j.snb.2015.12.028

58. Yi, W.; Li, Z.; Dong, C.; Li, H.W.; Li, J. Electrochemical detection of chloramphenicol using palladium nanoparticles decorated reduced graphene oxide. *Microchem. J.* **2019**, *148*, 774–783. https://doi.org/10.1016/j.microc.2019.05.049

77. Mei, J.; Leung, N.L.C.; Kwok, R.T.K.; Lam, J.W.Y.; Tang, B.Z. Aggregation-induced emission: Together we shine, united we soar! *Chem. Rev.* **2015**, *115*, 11718–11940. https://doi.org/10.1021/acs.chemrev.5b00263

90. Liu, S.; Bai, J.; Huo, Y.; Ning, B.; Peng, Y.; Li, S.; Han, D.; Kang, W.; Gao, Z. A zirconium-porphyrin MOF-based ratiometric fluorescent biosensor for rapid and ultrasensitive detection of chloramphenicol. *Biosens Bioelectron.* **2019**, *149*, 111801. https://doi.org/10.1016/j.bios.2019.111801.

91. Li, J.; Qu, L.; Li, H.; Zhao, L.; Chen, T.; Liu, J.; Gao, Y.; Pan, H. An electrochemical aptasensor for the detection of chloramphenicol based on ultra-small Au-inserted hollow PCN-222 MOF. *Microchim Acta* **2023**, *190*, 366. https://doi.org/10.1007/s00604-023-05949-y.

92. Wang, K.P.; Zhang, Y.C.; Zhang, X.; Shen, L. Green preparation of chlorine-doped graphene and its application in electrochemical sensor for chloramphenicol detection. *SN Appl. Sci.* **2019**, *1*, 157. https://doi.org/10.1007/s42452-019-0174-4.

93. Umamaheswari, R.; Manavalan, S.; Chen, S.M.; Govindasamy, M.; Chen, T.W.; Maiyalagan, T. Microwave-assisted synthesis of europium(III) oxide decorated reduced graphene oxide nanocomposite for detection of chloramphenicol in food samples. *Compos B Eng.* **2018**, *161*, 29–36. https://doi.org/10.1016/j.compositesb.2018.10.043.

94. Jayan, H.; Sun, D.W.; Pu, H.; Wei, Q. Mesoporous silica coated core-shell nanoparticles substrate for size-selective SERS detection of chloramphenicol. *Spectrochim. Acta A Mol. Biomol.* **2023**, *284*, 121817. https://doi.org/10.1016/j.saa.2022.121817.

95. Liu, S.; Lai, G.; Zhang, H.; Yu, A. Amperometric aptasensing of chloramphenicol at a glassy carbon electrode modified with a nanocomposite consisting of graphene and silver nanoparticles. *Microchim. Acta,* **2017**, *184,* 1445–1451. https://doi.org/10.1007/s00604-017-2138-y.

96. Gao, L.L.; Li, S.P.; Wang, Y.; Wu, W.N.; Zhao, X.L.; Li, H.J.; Xu, Z.H. Quinoline-based hydrazone for the colorimetric detection of Co2+ and fluorescence turn-on response of Zn2+. *Spectrochim. Acta A Mol. Biomol. Spectrosc.* **2020**, *230*, 118025. https://doi.org/10.1016/j.saa.2020.118025.

97. Shi, L.; Chang, D.; Zhang, G.; Zhang, C.; Zhang, Y.; Dong, C.; Chu, L.; Shuang, S. Co^{2+} detection, cell imaging, and temperature sensing based on excitation-independent green-fluorescent N-doped carbon dots. *RSC Advances* **2019**, *9*, 41361–41367. https://doi.org/10.1039/c9ra09405a.

98. Ismail, H.; Ahmad, M.N.; Normaya, E. A highly sensitive and selective thiosemicarbazone chemosensor for detection of Co2+ in aqueous environments using RSM and TD/DFT approaches. *Sci Rep.* **2021**, *11*, 20963. https://doi.org/10.1038/s41598-021-00264-z.

99. Temel, N.K.; Çöpür, M. Determination of trace cobalt (II) in spices samples by ultrasonic assisted cloud point extraction with spectrophotometry. *J. Mol. Struct.* **2023**, *1284*, 135433. https://doi.org/10.1016/j.molstruc.2023.135433.

100. Tavallali, H.; Deilamy-Rad, G.; Parhami, A.; Mousavi, S.Z. A novel development of dithizone as a dual-analyte colorimetric chemosensor: Detection and determination of cyanide and cobalt (II) ions in dimethyl sulfoxide/water media with biological applications. *J. Photochem. Photobiol. B Biol.* **2013**, *125*, 121–130. https://doi.org/10.1016/j.jphotobiol.2013.05.013.

101. Gore, A.H.; Gunjal, D.B.; Kokate, M.R.; Sudarsan, V.; Anbhule, P.V.; Patil, S.R.; Kolekar, G.B. Highly Selective and Sensitive Recognition of Cobalt(II) Ions Directly in Aqueous Solution Using Carboxyl-Functionalized CdS Quantum Dots as a Naked Eye Colorimetric Probe: Applications to Environmental Analysis. *ACS Appl Mater Interfaces.* **2012**, *4*, 5217–5226. https://doi.org/10.1021/am301136q.

102. Xie, Z.; Zhang, F.; Pan, Y. Chemiluminescence detection of trace amounts of cobalt (II) with the 2,6,7-trihydroxy-9-(4′-chlorophenyl)-3-fluorone–hydrogen peroxide–cetyltrimethylammonium bromide system. *The Analyst* **1998**, *123*, 273–275. https://doi.org/10.1039/a705880b.

103. Farahani, T.Z.; Bagherian, G.; Chamjangali, M.A.; Ashrafi, M. On-line determination of the trace amount of cobalt(II) in real samples by flame atomic absorption spectrometry method after pre-concentration by modified polyvinyl chloride. *SN Appl. Sci.* **2023,** *5,* 183. https://doi.org/10.1007/s42452-023-05402-y.

104. Antohe, I. Cobalt Ions Detection Using an Evanescent Wave Optical Fiber Sensor. *Rom. Rep. Phys.* **2022**, *74*, 505.

105. Talio, M.C.; Alesso, M.; Acosta, M.G.; Acosta, M.; Fernández, L.P. Sequential determination of lead and cobalt in tap water and foods samples by fluorescence. *Talanta* **2014**, *127*, 244–249. https://doi.org/10.1016/j.talanta.2014.04.020.

106. Zafer, M.; Keskin, C.S.; Özdemir, A. Highly sensitive determination of Co(II) ions in solutions by using modified silver nanoparticles. *Spectrochim. Acta A Mol. Biomol. Spectrosc.* **2020**, *239*, 118487. https://doi.org/10.1016/j.saa.2020.118487.

107. El-Feky, H.H.; Amin, S.A.; Moustafa, E.M.I. Utilization of a plasticized PVC optical sensor for the selective and efficient detection of cobalt (II) in environmental samples. *RSC Adv.* **2022,** *12*, 18431–18440. https://doi.org/10.1039/D2RA03129A.

108. Ojeda, C.B.; Rojas, F.S.; Pavón, J.M.C. Determination of Cobalt in Food, Environmental and Water Samples with Preconcentration by DispersiveLiquid-Liquid Microextraction. *Am. J. Anal. Chem.* **2012**, *3*, 125–130. https://doi.org/10.4236/ajac.2012.32018.

109. Wang, S.; Meng, S.; Guo, Y. Cloud Point Extraction for the Determination of Trace Amounts of Cobalt in Water and Food Samples by Flame Atomic Absorption Spectrometry. *J. Spectrosc.* **2013**, *2013*, 735702. https://doi.org/10.1155/2013/735702.

110. Tekin, Z.; Unutkan, T.; Erulaş, F.; Bakırdere, E.G.; Bakırdere, S. A green, accurate and sensitive analytical method based on vortex assisted deep eutectic solvent-liquid phase microextraction for the determination of cobalt by slotted quartz tube flame atomic absorption spectrometry. *Food Chem.* **2019**, *310*, 125825. https://doi.org/10.1016/j.foodchem.2019.125825.

111. Liu, Y.L.; Yang, L.; Li, L.; Guo, Y.-Q.; Pang, X.X.; Li, P.; Ye, F.; Fu, Y. A New Fluorescent Chemosensor for Cobalt (II) Ions in Living Cells Based on 1,8-Naphthalimide. *Mol.* **2019**, *24*, 3093. https://doi.org/10.3390/molecules24173093.

112. Kang, S.M.; Jang, S.C.; Kim, G.; Lee, C.S.; Huh, Y.; Roh, C. A Rapid in Situ Colorimetric Assay for Cobalt Detection by the Naked Eye. *Sensors* **2016**, *16*, 626. https://doi.org/10.3390/s16050626.

113. Alomar, T.S.; Habila, M.A.; AlMasoud, N.; Alothman, Z.A.; Sheikh, M.; Soylak, M. Biomass-derived adsorbent for dispersive solid-phase extraction of Cr (III), Fe (III), Co (II) and Ni (II) from food samples prior to ICP-MS detection. *Appl. Sci.* **2021**, *11*, 7792. https://doi.org/10.3390/app11177792.

114. Khalil, S.; El-Sharnouby, M. Construction of a Highly Selective Membrane Sensor for the Determination of Cobalt (II) Ions. *Chemosensors* **2021**, *9*, 86. https://doi.org/10.3390/chemosensors9050086.

115. Santos, L.B.; Assis, R.D.S.D.; Silva, U.N.; Lemos, V.A. Switchable-hydrophilicity solvent-based liquid-phase microextraction in an on-line system: Cobalt determination in food and water samples. *Talanta* **2022**, *238*, 123038. https://doi.org/10.1016/j.talanta.2021.123038.

116. Fagadar-Cosma, E.; Fagadar-Cosma, G.; Vasile, M.; Enache, C. Synthesis, Spectroscopic and Self-Assembling Characterization of Novel Photoactive Mixed Aryl-Substituted Porphyrin. *Curr. Org. Chem.* **2012**, *16*, 931–941. https://doi.org/10.2174/138527212800194755.

117. Arnold, J. *The Porphyrin Handbook*; Kadish, K.M., Smith, K.M., Guilard, R., Eds.; Academic Press: San Diego, CA, USA, 2000; Volume 3, pp. 113–127.

118. Stuzhin, P.A. Theoretical AM1 study of acidity of porphyrins, azaporphyrins and porphyrazines. *J Porphyr Phthalocyanines* **2003**, *7*, 813–832. https://doi.org/10.1142/s1088424603001014.

119. Gomes, A.T.P.C.; Neves, M.G.P.M.S.; Cavaleiro, J.A.S. Cancer, Photodynamic Therapy and Porphyrin-Type Derivatives. *An. Acad. Bras. Cienc.* **2018**, *90*, 993–1026. https://doi.org/10.1590/0001-3765201820170811 728.

120. Callaghan, S.; Senge, M.O. The good, the bad, and the ugly–controlling singlet oxygen through design of photosensitizers and delivery systems for photodynamic therapy. *Photochem. Photobiol. Sci.* **2018**, *14*, 1490-1514. https://doi.org/10.1039/c8pp00008e.

121. Redl, F.X.; Lutz, M.; Daub, J.; Chemistry of Porphyrin-Appended Cellulose Strands with a Helical Structure: Spectroscopy, Electrochemistry, and in situ Circular Dichroism Spectroelectrochemistry. *Chem. Eur. J.* **2001**, *7* 5350–5358. https://doi.org/10.1002/1521-3765(20011217)7:24%3C5350::AID-CHEM5350%3E3.0.CO;2-F.

122. Rodríguez-Morgade, M.S.; de la Torre, G.; Torres, T. *The Porphyrin Handbook*; Kadish, K.M., Smith, K.M., Guilard, R., Eds.; Academic Press: San Diego, CA, USA, 2003; Volume 15, pp. 125–159.

123. Dmitrieva, O.A.; Ivanova, Y.B.; Semeikin, A.S.; Mamardashvili, N.Z. Fluorescence properties and quantum-chemical modeling of tert-butyl-substituted porphyrazines: Structural and ionization effect. *Spectrochim. Acta A Mol. Biomol. Spectrosc.* **2020**, *240*, 118601. https://doi.org/10.1016/j.saa.2020.118601.
